# Magnesium-Based Materials for Hydrogen Storage—A Scope Review

**DOI:** 10.3390/ma13183993

**Published:** 2020-09-09

**Authors:** Agata Baran, Marek Polański

**Affiliations:** Department of Functional Materials and Hydrogen Technology, Military University of Technology, Kaliskiego 2 Street, 00-908 Warsaw, Poland; agata.baran@wat.edu.pl

**Keywords:** hydrogen storage, solid-state hydrogen storage, mechanochemical synthesis, ball milling, mechanical alloying, reactive ball milling, magnesium hydride, magnesium-based hydrides, magnesium ternary hydrides, Mg_2_FeH_6_, Mg_2_NiH_4_, Mg_2_CoH_5_

## Abstract

Magnesium hydride and selected magnesium-based ternary hydride (Mg_2_FeH_6_, Mg_2_NiH_4,_ and Mg_2_CoH_5_) syntheses and modification methods, as well as the properties of the obtained materials, which are modified mostly by mechanical synthesis or milling, are reviewed in this work. The roles of selected additives (oxides, halides, and intermetallics), nanostructurization, polymorphic transformations, and cyclic stability are described. Despite the many years of investigations related to these hydrides and the significant number of different additives used, there are still many unknown factors that affect their hydrogen storage properties, reaction yield, and stability. The described compounds seem to be extremely interesting from a theoretical point of view. However, their practical application still remains debatable.

## 1. Introduction

In recent decades, the need for new energy carriers has increased [1]. High and still growing worldwide energy consumption [2] (mainly based on fossil fuels) has greatly influenced irreversible global climate change [3]. Thus, the need to expand to new, efficient, and reliable sources of energy has become essential. The greatest challenge is to transform our carbon-based economy to a carbon-free economy [4]. Renewable energy sources are candidates to replace fossil fuels. Unfortunately, they are, to an extent, limited and unevenly distributed. The full exploitation of these energy sources requires an efficient energy carrier and storage system. Hydrogen has been considered for a long time to solve the problems mentioned above and has influenced broad technological investigations involving issues associated with its production, storage, and application [5]. Hydrogen is an almost ideal energy carrier that is considered to be a clean fuel and has the largest gravimetric density of all known chemical substances (~3 times higher than that of gasoline) [6]. Moreover, hydrogen is the most abundant element (~15 mol%) on the surface of the Earth (found in water, biomass and fossil fuels) [7]. The European Commission describes hydrogen as a new energy carrier with “a great potential for clean, efficient power in stationary, portable, and transport applications” [8]. Hydrogen is an important element of the energy chain of alternative energy sources (such as solar, wind, ocean, and geothermal) because of its environmental compatibility and efficiency and because it is thought to be adequate for mobile applications [9]. Hydrogen-fueled transportation started with the Apollo 11 mission to the moon and has continued until today, with hydrogen fuel cell vehicles, trains, and ferries [8]. A hydrogen economy has been developed over the last decades, but despite the billions of dollars spent, the storage problem is still a challenge. Therefore, it is necessary to find a reliable and effective method for hydrogen storage because all of the known solutions have advantages and disadvantages [10,11].

Solid-state hydrogen storage is a promising option when considering some of its unique features (such as heat evolution during loading and low-pressure filling). This solution is characterized by having the highest volumetric density (higher than that of liquid hydrogen) and, when properly used, is relatively safe [12]. On the other hand, solid-state hydrogen storage is characterized by a rather low energy density per mass unit temperature even without considering the material itself instead of the mass of the system [13]. Solid-state vessels usually do not need to work under high pressure, and hydrogen is released in an endothermic reaction, which, depending on the conditions, material and vessel size, may take from minutes to days [14] due to the extremely low heat conductivity of hydrides, which in real systems remains in the effective range of insulators (<1 W/m/K), even those that are metallic alloys [15]. Not considering low-temperature adsorption on porous materials, solid-state hydrogen storage is based on metal hydrides, intermetallic hydrides, and complex hydrides [16]. These materials are believed to be a safe, risk-free solution and have recently become an alternative to conventional methods [17]. However, many safety issues still have to be solved, as dealing with kilograms of usually very pyrophoric powders can be an issue from a legal point of view in addition to causing engineering problems. There is an abundance of solid-state hydrogen storage materials, but currently, only some are of great importance, e.g., intermetallic AB_5_-, AB_2_-, and AB-type alloys, which are practically used and commercially sold. However, none of the above materials stores more than 2% hydrogen by weight, which is a very serious drawback in potential mobile applications [18]. On the other hand, most solid-state hydrogen storage materials offer a higher volumetric hydrogen density than liquid hydrogen (at least when lattice density is considered).

One of the most investigated types of materials (mainly due to their relatively high gravimetric capacity) is a group of magnesium-based hydrides, including pure magnesium. Magnesium is a low-density, relatively inexpensive and highly abundant (in the form of different compounds in Earth’s crust) metal [19]. In its pure form, magnesium can absorb hydrogen (preferably at >400 °C) at up to 7.6 wt.%, but it has low stability (readily reacting with oxygen, for example) and low hydrogen absorption/desorption kinetics [16]. To date, substantial efforts have been made to examine the optimum desorption pressure and temperature for reactions to take place [20], to improve (or at least better understand) poor cycle life and slow kinetics by doping with catalysts [21,22], and to investigate destabilization [23,24,25,26], crystallite size reduction [27,28,29] and even heavy-ion irradiation effects [30]. Some known compounds (Mg_2_NiH_4_, Mg_2_CoH_5_, and Mg_2_FeH_6_) appear to be interesting alternatives to pure magnesium hydride, creating a compromise between hydrogen capacity by volume or mass and different equilibrium pressures, stability, and costs. The largest hydrogen capacities (by volume) have been observed with compounds of the form Mg_2_TMH_x_ (TM = Ni, Co, Fe), which also have very high gravimetric capacities (3.6–5.6%) [31,32,33,34,35,36,37,38,39,40,41,42,43,44,45]. Among the different methods of energy storage, metal hydride-based materials are also ideal candidates for the future storage of thermal energy due to their capability to store and release substantial amounts of heat at high temperatures. This process can be driven only by slight pressure changes [46]. The successful attempts to synthesize such materials from magnesium and steel (even stainless) waste (scrap) [47,48,49,50] suggest that there is a real chance of implementing them as a low-cost storage solution.

A mechanochemical synthesis method that utilizes ball-milled magnesium hydride (with and without additives) and alloying metals has been employed to produce potential hydrogen storage materials with high efficiency. This method of synthesis allows not only the introduction of defects to the material and an increase in hydrogen diffusion but also the direct synthesis of compounds and doping of the materials with catalysts. The number of scientific papers related to magnesium hydride and related compounds since 1951, when magnesium hydride synthesis from pure elements [51] was presented, can easily be counted in the thousands. Recently, several review papers were published in this field, which summarized the issues related to hydrogen storage in this material group. Due to the ongoing investigation of a large number of issues related to these materials, from synthesis method efficiency, reaction mechanisms, and structural characterization to the improvement of reaction kinetics, destabilization, sintering problems, safety issues, scaling up and heat exchange, it would be nearly impossible to prepare one review to cover all such subjects. For example, Jain et al. [52] briefly presented magnesium as a hydrogen storage medium. However, they provided only a brief overview and some kinetic problems, while the formation of ternary hydrides was presented as alloying with transition elements only. Additionally, Huot et al. [18], in their very successful work, described the basic properties of Mg as well as Mg_2_NiH_4_, Mg_2_FeH_6_, and Mg_2_CoH_5_. However, because this work had a very broad scope and was focused on mechanochemistry, the authors did not provide many details about reaction pathways or other modifications. A very detailed work was provided by Webb [21], who focused mainly on magnesium hydride and additives. However, ternary hydrides were not described. Crivello et al. [53] reviewed the problems related to MgH_2_, including theoretical explanations and DFT modeling of structure and phase diagrams, methods of MgH_2_ processing, including not only ball milling (BM), but also more unique techniques (such as severe plastic deformation and plasma-enhanced synthesis), and the behavior of magnesium pellets. In their second review paper [54], they focused mostly on the destabilization reactions and use of alloying elements leading to drastic changes in reaction enthalpy. The effects of both catalytic and destabilizing additives on magnesium hydride were summarized in the review by Wang and Wang [55]. Despite the compactness of the paper, the authors managed to present different types of additives, including organometallic additives. The review of Zhang et al. [56] covered a similar scope but emphasized the nanostructured nature of both additives and substrates. Problems related to nanoconfinement and nanocatalysts were described in detail in their work. A significant number of magnesium-based materials were presented and described in work by Yartys et al. [16], together with their modification, their cycling stability, and a historical overview. An important summary of the recent progress in the field of Mg-Fe-H and Mg-Co-H systems was given by Puszkiel et al. [57], but neither pure magnesium-based materials nor Mg-Ni systems were described in their book chapter.

Each of the recently published reviews addressed magnesium-related problems with great care, putting stress on different aspects. In this scope review, we have chosen magnesium, Mg_2_NiH_4_, Mg_2_FeH_6_ and Mg_2_CoH_5_ (which can sometimes be treated as MgH_2_ derivatives) since in many cases, these materials coexist or can successfully replace each other in chosen applications due only slight differences in properties. They are also characterized by similar thermodynamics and mechanisms of formation despite having significant differences in behavior and stability. Furthermore, successful replacement of certain elements (Fe/Ni/Co) has been performed, resulting in materials with mixed properties [50,58]. The real problem is that the large amount of available data “on the market”, despite the apparent simplicity of the systems, makes it difficult to distinguish reliable research that expresses new ideas from research that unwittingly replicates old ideas that have already been forgotten. Some of the new papers present physically impossible results that, despite having been published, should be forgotten and not mentioned anymore. In this work, we have focused on the material group with which we have personal experience. On the basis of this experience, we sometimes narratively present some of the issues related to magnesium hydride and the mentioned ternary hydrides, putting stress on the results and issues of great importance while in some cases omitting data that have been published but seem to be redundant with the chosen papers. The main aim of publishing this paper is to help researchers starting work in this field to gain basic knowledge about magnesium-based hydrogen storage materials and to present results that provide a basic overview of the current state of the art in this field. However, it must be strongly stressed that several other important subjects are outside of the scope of this work.

## 2. Magnesium Hydride

**Figure 1:**
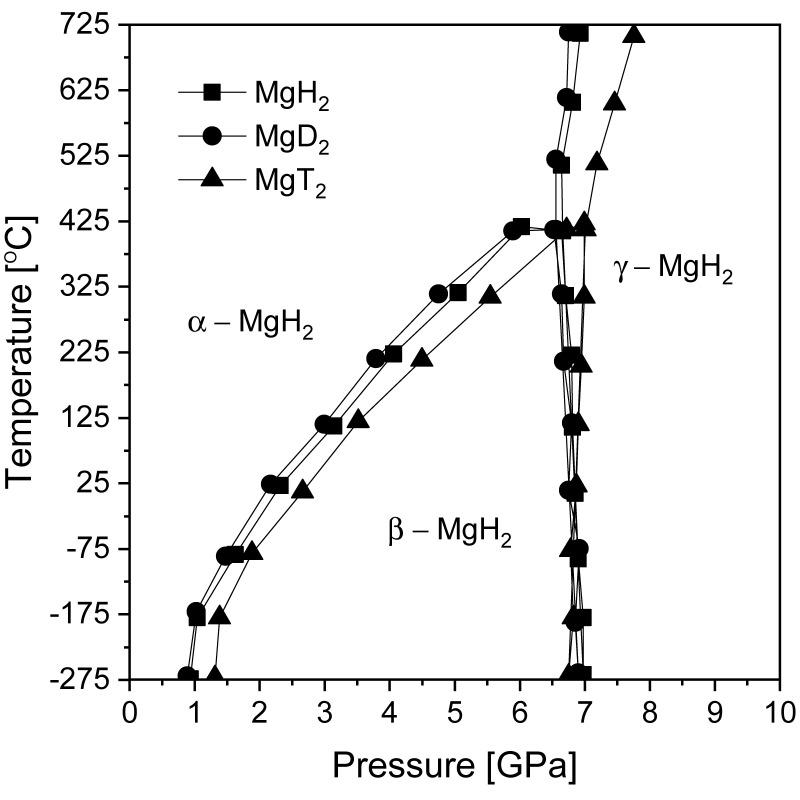
Pressure-temperature (P–T) phase diagrams for three studied isotopic analogs of magnesium dihydride: MgH_2_, MgD_2_, and MgT_2_. The graph was prepared based on [56,59].

Magnesium hydride has several known polymorphs that are thermodynamically stable at different temperatures and pressures, which was experimentally verified by Bastide et al. [59] and later shown by modeling work. Figure 1 shows pressure-temperature phase diagrams for magnesium hydride (and its isotopic analogs) calculated by Moser et al. [60]. Knowledge about the stability of MgH_2_ phases is crucial in understanding the formation of its polymorphs and may be important in the interpretation of experimental data, especially for ball-milled samples. Magnesium hydride exists as an α-MgH_2_ (with a rutile structure) phase under ambient conditions [61]. Changing the temperature and pressure leads to a phase transformation. High pressure is needed to change α-MgH_2_ (TiO_2_) to β- and γ-MgH_2_ at low temperatures. The transformation of the β-MgH_2_ phase (with a modified CaF_2_ structure) is possible only at temperatures below 700 °C. Above that temperature, direct transformation from α-MgH_2_ to γ-MgH_2_ (with an orthorhombic structure similar to that of α–PbO_2_) occurs. From a practical point of view, in hydrogen storage research, only two polymorphs are important. One polymorph (the TiO_2_ structure) is stable under ambient conditions, and the second, which is metastable (the PbO_2_-type structure), appears in magnesium hydride processed by BM for a long time [62] due to the high pressure generated by the collisions of balls with the milled material and cylinder walls. The γ phase (γ-MgH_2_) is a high-pressure polymorphic form of the β-MgH_2_ phase [59]. It is important to note that, over time, and for unknown reasons, the phase names originally given by Bastide et al. [59] changed, which may cause significant confusion to readers. What was originally named the α phase (TiO_2_-type structure) is currently called the β phase in most current papers. Fortunately, the γ phase still describes the originally named γ phase.

**Figure 2:**
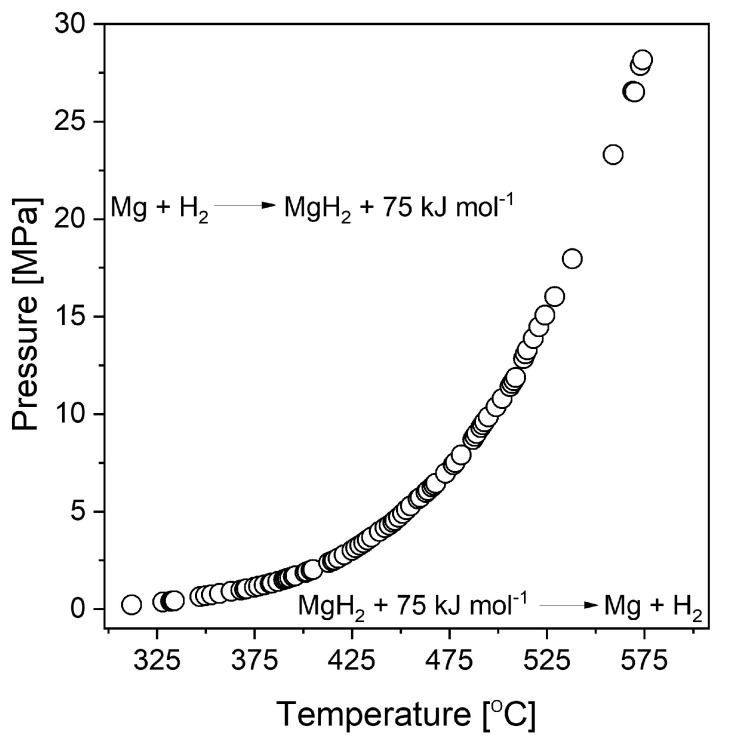
Temperature dependence of the MgH_2_ dissociation pressure. The graph was prepared based on data from Stampfer et al. [20].

Magnesium hydride (MgH_2_) is widely investigated due to its relatively high gravimetric and volumetric densities (ρ_m_ = 7.6 wt.% H and ρ_V_ = 0.11 kg H/dm^3^, respectively). Its dissociation enthalpy was first measured by Stampfer et al. [20] based on decomposition pressure measurements between 314 and 576 °C. Due to its high enthalpy of formation, MgH_2_ is considered a stable hydride. Figure 2 presents the dependence of the decomposition plateau pressure on temperature for magnesium hydride. The graph was prepared based on data obtained from Stampfer’s measurements [20]. From this graph, a temperature of 285 °C was estimated to be required to desorb hydrogen at a pressure of one bar (0.1 MPa) (usually, desorption at that temperature is very difficult to achieve in noncatalyzed systems due to very slow kinetics).

**Figure 3:**
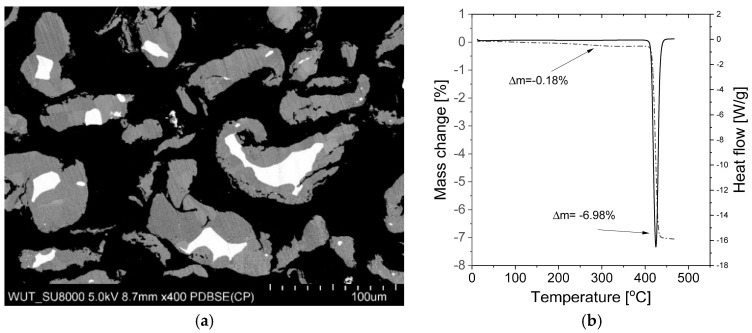
(**a**) Cross-section of commercially available magnesium hydride particles with visible white magnesium cores; MgH_2_ supplied by Alfa Aesar (photo taken at Warsaw University of Technology by dr Tomasz Płociński). (**b**) TGA/DSC curve for commercial MgH_2_.

The high thermodynamic stability of MgH_2_ is a serious drawback, but technological and practical issues also make studies on this material difficult. Even now, tens of years after the first synthesis was achieved in 1951 by Wiberg et al. [51], it is difficult to find commercial magnesium hydride with a purity of more than 90% (despite the official specifications given by manufacturers). Instead, magnesium hydride is usually a mixture of magnesium hydride, magnesium metal, and magnesium hydroxide contamination. Moreover, the hydroxide is usually present in the form of an amorphous layer on the surface of the particles, making it very difficult to observe (for example, by X-ray diffraction (XRD)), while magnesium is present in the core of the particles. These observations result in the measured amount of unreacted metal being low due to the weak diffraction obtained from the “hidden” magnesium. Figure 3a shows a cross-section of commercially available magnesium hydride particles. The proper choice of scanning electron microscopy (SEM) parameters and ion milling allows the differences in the atomic mass densities of MgH_2_ and Mg to be observed, which are usually not easy to observe. Notably, magnesium metal in its pure form (which likely does not react with hydrogen during the synthesis process) can be found inside larger particles, and thus its amount can hardly be called negligible, despite the fact that the analyzed sample was a commercial product (Alfa Aesar, Haverhill, MA, USA). The presence of hydroxide, on the other hand, can usually be observed only with the use of thermogravimetric analysis (TGA). An investigative analysis performed in our laboratory on a commercially available material showed that the significant mass losses (~0.2%) that were observed were related to the release of -OH groups from hydroxides, while almost no noticeable heat effect could be seen from the differential scanning calorimetry (DSC) curve obtained at the same time (Figure 3b). Also the maximum capacity is far from the theoretical one, and the show example is one of the best results obtained for commercially available MgH_2_. In practice, it is very difficult to convert magnesium to magnesium hydride below 350 °C, even when the magnesium is in the form of very fine powder (again, due to slow kinetics). In the case of coarser particles (a coarse powder or turnings), such conversion is almost impossible, even at a much higher temperature and pressure, due to the formation of a hydride layer on the outside, preventing the reaction from occurring throughout the whole volume of the particle [63,64]. Thus, a two-step synthesis was developed to transform the remaining sample volume into the metal hydride. The main scientific goal was to decrease the practical absorption and desorption temperatures while maintaining the same volumetric and gravimetric densities.

Much effort has been made to overcome problems associated with synthesis outcomes, which are related to the previously discussed poor reaction kinetics of hydrides. The main approach for changing the sorption behavior of a hydride without decreasing its hydrogen capacity involves decreasing the grain or crystallite size. The grain size effect and the role of surface modifications (surface activity, oxide layer penetration, diffusion rate of hydrogen, and mobility of metal-hydride interfaces) on the sorption characteristics of hydrides were shown for the first time by Zaluska et al. [28]. The conducted research was inspired by previous attempts related to powder absorption [64,65]. The fabricated magnesium powders had a similar particle size, but the grain size (or better, crystallite size) inside the particles was different. Thus, a nanocrystalline structure combined with surface modification was shown to greatly improve the hydrogenation and dehydrogenation rates. Over time, more research has been devoted to this issue [27,29,62,66]. Following the idea of lowering the size of crystallites and particles, Nielsen et al. investigated MgH_2_ nanocluster confinement within nanoporous aerogel scaffolds [67]. Additionally, a broad review was written to organize the knowledge about nanoconfined hydrides for energy storage [68]. Later, Kim et al., in their theoretical work [69] based on the Wulff construction, predicted the influence of nanoparticle size on the thermodynamics of hydrogen release. Their research suggested that destabilization of the hydride phase was possible with a decrease in particle size. In most of the considered cases, the desorption temperature should decrease slightly upon reducing the particle size, but these changes were relatively small in comparison with the properties of the bulk material. Practical attempts were presented by Paskevicius et al. [70] and Zhao-Karger [71]. A decrease in particle size (down to the nanometer scale) led to a decrease in the hydrogenation/dehydrogenation energy (lower values of enthalpy and entropy) for the nanoconfined system compared with that of the bulk material. As a result of thermodynamic destabilization, the 0.1 MPa hydrogen equilibrium temperature decreased by ~6 °C or even 11 °C. Thus, the thermodynamics of smaller nanoparticles were dominated by changes in the enthalpy of the reaction. Hence, the temperature reduction was smaller than theoretically predicted [70]. This decrease in hydrogen desorption energy has been predicted to work only for relatively small clusters (MgH_2_ crystallite sizes of ~1.3 nm) [72]. It is worth mentioning that for particles, only the crystallite size, not the grain size, should be small. The thermodynamic stability of MgH_2_ with respect to that of Mg + H_2_ as a function of crystal grain size was investigated by Wagemans et al. [72]. Their calculations showed that MgH_2_ became less stable than Mg as the cluster size decreased. Small clusters needed less desorption energy, which led to a low hydrogen desorption temperature. Quantum Monte Carlo simulations were performed by Wu et al. [73] to verify experimental data and estimate the nanoparticle size with the most beneficial desorption temperature. Unfortunately, it turned out that explaining the experimentally observed nanoscale effects in metal hydrides required more information than just the cluster size. Therefore, the authors suggested that the specific chemical environment of the nanoparticles played a crucial role in terms of hydride destabilization. Recently, some experimental works confirmed this theoretical prediction after successful syntheses of MgH_2_ nanoclusters, e.g., by using an immiscible system (Mg-Ti) as a precursor [74]. Magnesium nanoclusters were also synthesized by Huang. However, the authors did not show a difference in the decomposition enthalpy (what should be expected), but rather focused on the decomposition kinetics [75].

### 2.1. MgH_2_ Synthesis/Mechanical Modifications

Mechanochemical synthesis or milling allows the production of magnesium hydride with or without additives [18,76] by reactive ball milling (RBM), which is basically BM in a hydrogen atmosphere, or by simply BM in an inert atmosphere, respectively. These are the most common techniques used for the production of many metal hydrides. In the case of RBM, the balls (the most commonly used balls are made of steel or tungsten carbide) are placed together with metallic particles in a pressurized vial of hydrogen, and their high-energy impact results in the fracture and cold-welding of the metallic particles while also reacting to hydrogen. BM in an inert atmosphere is conducted without pressurizing the vial with hydrogen. Thus, chemically produced magnesium hydride must be used for modification by BM. Both techniques help to reduce the particle and crystallite size and to induce γ phase (high-pressure polymorph) formation. BM requires the use of a proper combination of parameters, namely the type of ball mill, ball-to-powder ratio, milling time, speed, temperature, and hydrogen pressure [18,29,77]. Slight modification of even one parameter results in a material with different properties. All of these factors make a comparison of all published research complicated since, in most cases, there may not be even a single common parameter among different experiments. In many cases, the experiments are not described extensively enough to be replicated, but BM in general leads to an improvement in hydride properties (mainly a decrease in sorption temperature). This improvement is the reason why this method is still an attractive way of producing MgH_2_.

### 2.2. Ball Milling

**Figure 4:**
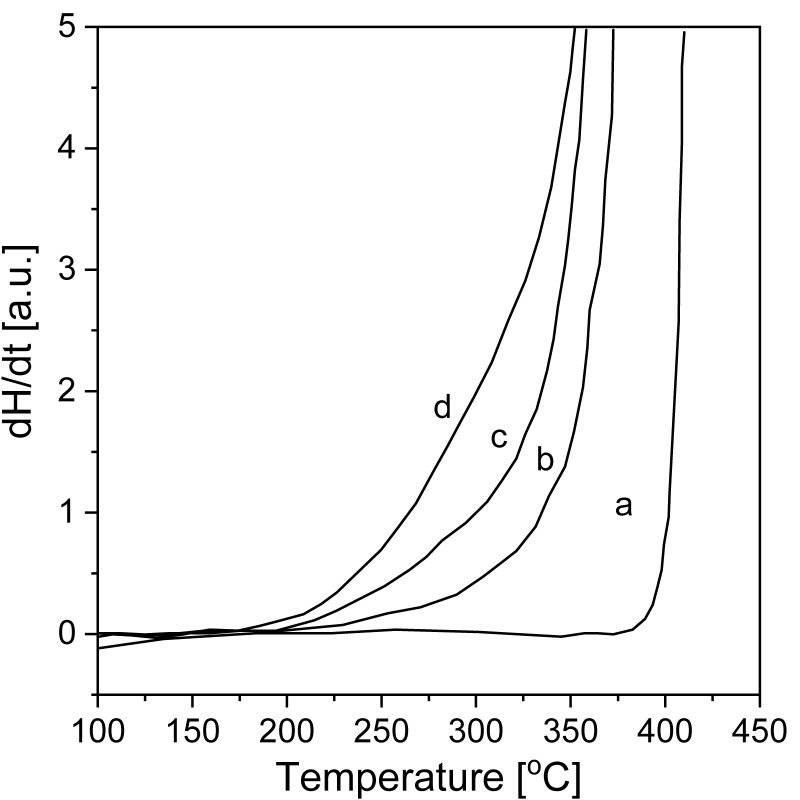
Onset sections of the DSC curves of MgH_2_ desorption after BM for different periods of time: (a) initial magnesium hydride, (b) after BM for 2 min, (c) after BM for 7 min, and (d) after BM for 9 min. The graph was prepared based on data from [81]. The DSC plots were obtained with a heating rate of 40 °C/min.

The dissociation of hydrogen molecules occurs on the surface of the metal. For this reason, in the first stages, absorption is determined by the hydrogen dissociation activity [78]. It is known that pure Mg chemisorption is rather slow [79]. Moreover, as the reaction progresses, a hydride layer grows on the metal surface, and the ability to diffuse through this layer becomes limited [64,78]. BM helps to improve the reaction. The surface area increases because micro- or even nanostructures form, and defects are introduced. A high number of defects provides different hydriding properties and behaviors [28]. Therefore, changing the alloy composition, surface features, or technological parameters during BM helps to control the material properties, such as the reaction kinetics and storage capacity [80]. The first attempt of pure MgH_2_ synthesis by BM was conducted by Strom-Olsen with Zaluski and Zaluska [81]. The research was inspired by previous synthesis attempts (mostly involving the synthesis of Mg_2_Ni, but also MgH_2_, by RBM) [82,83,84]. This synthesis was a two-step method: first, magnesium powder was ball milled in argon, and then, the material was hydrogenated in a gas titration system at a hydrogen pressure of 1 MPa and 310 °C. Improvements in powder morphology and surface activity were noticed during hydrogenation. Moreover, no traces of unreacted magnesium were found. The enhanced kinetics (the first dehydrogenation occurred between 270 and 280 °C, and a hydrogen capacity of almost 7 wt.% was reached during the subsequent hydrogenation) remained unaltered even after 15 cycles. The results showed that with changes in milling time, the onset temperatures of desorption significantly decreased. The onset of desorption from the hydride can be seen in the DSC graphs of measurements obtained at a heating rate of 40 °C/min (Figure 4). Significant differences are evident even for such high heating rates. Upon reducing the average crystallite size (indicated by Bragg peak broadening in XRD plots), the desorption energy decreased, and the desorption temperature dropped by as much as 100 °C. Moreover, the desorption peak shifted by approximately 30–50 °C.

Using pure Mg for hydrogen storage has drawbacks—the material needs to be activated. To perform initial hydrogenation, the Mg must be exposed to hydrogen at a higher temperature and pressure than is required for subsequent normal operation. Nevertheless, the absorption and desorption kinetics can still be rather slow. Since modifying the magnesium hydride decomposition enthalpy is nearly impossible (except in the case of so-called destabilization, which will be described later), the main goal is to improve the decomposition and formation kinetics of MgH_2_. Fast hydrogenation kinetics directly lead to the possibility of a short refilling time for hydrogen storage tanks (if proper heat exchange conditions are fulfilled). Therefore, some research has concentrated on metal additives, and several attempts have been made to study the influence of metal oxides or additives on hydrides. Studies have shown the effects of using both metal oxides and additives. Table 1 presents selected research results and the basic sorption properties of BM-synthesized Mg-based hydrides. From the studies presented below, it is clear that the reaction kinetics have improved as a result of using different catalysts. Hence, the desorption temperatures have decreased. What is even more clear from the table is that the experimental conditions used in the literature are very different from each other. This variation makes it very difficult to compare reported values, practically making a comparison impossible other than just qualitatively. The observed maximum hydrogen capacities should also be treated very carefully, given that the maximum capacities range from 3.5% to 7% for no real physical reason, assuming that the amount of catalyst was not 50% by weight in some cases. A deeper look at the experimental conditions used in the selected research will reveal further differences in sample mass, gas purities, etc.

#### 2.2.1. Nanostructurization

It was shown in [91] that milling brittle MgH_2_ was more effective than milling pure magnesium powder (which is relatively ductile) due to combining a nanocrystalline structure with a high surface area. It was observed that mechanical deformation produced similar structural transformations to those obtained with high static pressure (in the range of 8 GPa). The hydrogen absorption rate of Mg-based alloys also increased with milling time. The examined material absorbed 7 wt.% H_2_ at 300 °C, and the same amount was desorbed at 350 °C (within 400 and 600 s, respectively).

**Figure 5:**
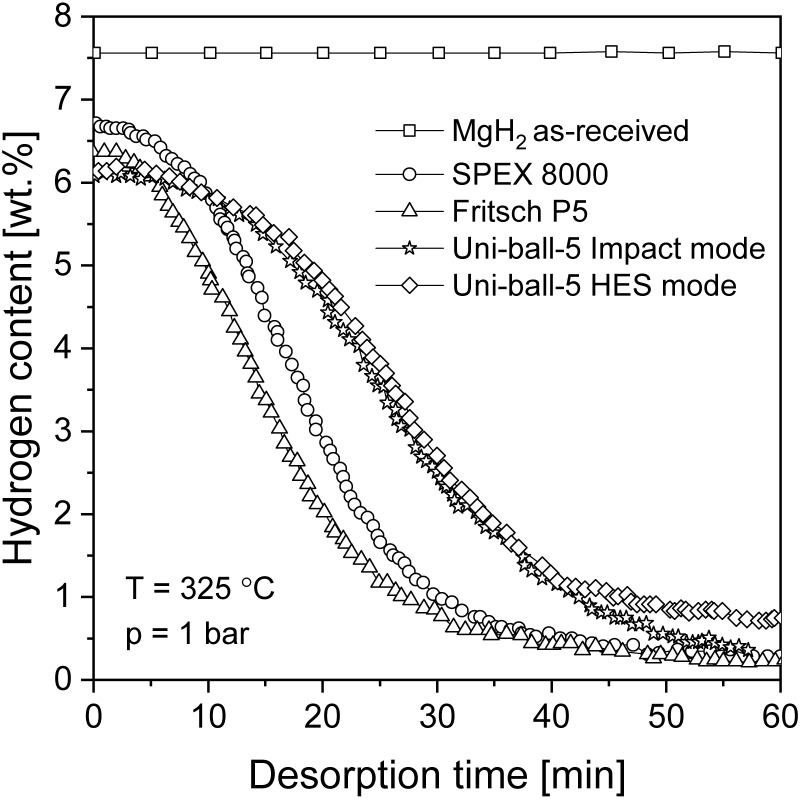
Desorption kinetics of nanocrystalline MgH_2_ ball milled for 20 h in different types of ball mills. The graph is based on [92].

In terms of nanostructurization conducted by mechanochemical methods, the influence of milling equipment on hydrogen sorption properties is crucial. It was proven in [92] that there was no significant difference between MgH_2_ samples milled in commonly used ball mills but that planetary mills appeared to be more productive than other types of mills (Figure 5). BM led to an improvement in the absorption and desorption kinetics, with only a small difference observed in the maximum hydrogen capacity when comparing different mills. It was suggested that high-energy BM had a great impact on hydriding/dehydriding properties due to the effect of reducing the particle and crystallite size (increasing the specific surface area), even when BM was performed in an argon atmosphere.

**Figure 6:**
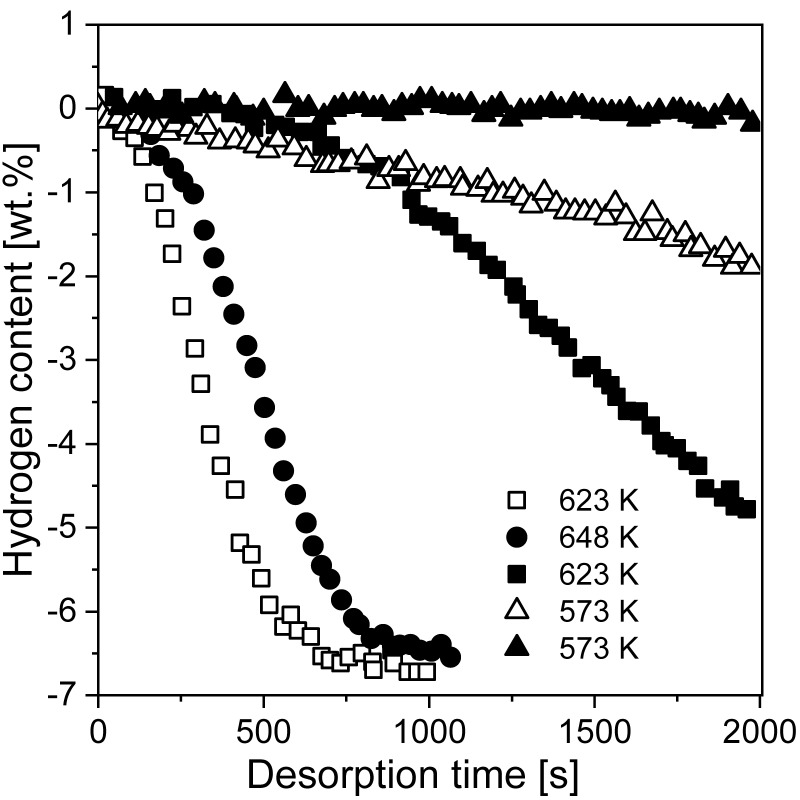
Hydrogen desorption curves of unmilled (solid symbols) and ball-milled (open symbols) MgH_2_ at 0.015 MPa hydrogen pressure. The graph was plotted using data from [80].

Comparisons between milled and unmilled MgH_2_ were provided by Huot et al. [80] (Figure 6) and by Vitorri Antisari et al. [93]. The sorption kinetics were found to be much faster for milled samples than unmilled samples. Milled hydride had better reaction kinetics and a lower activation energy. The ball-milled MgH_2_ desorption temperature was 64 °C lower than that measured for the unmilled sample [80]. Both the absorption and desorption of ball-milled magnesium hydride occurred at low temperatures and were much more rapid than those of unmilled magnesium hydride. Additionally, a 10-fold increase in specific surface area was observed after the BM technique was introduced, which was related to the results obtained by Schulz et al. [92]. The BM sample fully absorbed hydrogen at 300 °C and desorbed hydrogen at 350 °C at a relatively high rate. On the other hand, no significant change in storage capacity and no influence of BM on the thermodynamic properties of the obtained hydride were noticed. Therefore, improved kinetics are connected with the introduction of defects, a small particle size, and an increased specific surface area. These results prove that BM has advantages over other techniques in terms of kinetics, but fortunately does not significantly change the storage capacity of the material.

#### 2.2.2. Metal Additives

It is well known that the introduction of even a small amount of metal additives can significantly improve the hydrogen absorption and desorption kinetics of magnesium. The most popular metal catalysts are transition metals, e.g., Al, Fe, Cu, Pd, Ni, V, Nb, Ti, Mn, and Cr (synthesized together with Mg by BM and RBM techniques) [85,94,95,96,97,98,99,100,101,102,103,104]. However, recently, some alkali metals have also been used [105]. Vitorri Antisari et al. [93] showed a correlation between experimental results and a model describing the role of Fe catalyst particles in the nucleation step in the MgH_2_ reaction. The addition of Fe through the use of BM caused a significant increase in particle density with nucleation at the metal particles and in the bulk material. The newly formed structural defects acted as nucleation sites, exhibiting increased nucleation rates with increasing defect density. Hence, the reaction mechanism remained unaffected, but the rate of hydrogen desorption changed, thus proving that a surface catalyst could accelerate the reaction process. The cycle stability of hydrogen absorption/desorption and the hydrogen desorption activation energy were proven to be influenced by a vanadium additive [97,106]. Liang et al. [85] synthesized MgH_2_-5 mol% TM (TM = Ti, V, Mn, Fe, Ni) and showed that MgH_2_ ball milled with these five transition metals possessed superior hydrogen storage properties in terms of reaction kinetics. The addition of Ti increased the hydrogen absorption rate. V, Fe, Mn, and Ni were also beneficial in terms of desorption properties. Thus, different elements were profitable in different temperature ranges (e.g., the MgH_2_-Ti composite exhibited good properties in the 250–300 °C range, and the range for MgH_2_-V extended up to 200 °C). Later, Liang et al. [107] proved that the superior hydrogen desorption properties were caused by vanadium particles and their strong affiliation with hydrogen. Pelletier et al. [108] found that during BM of a MgH_2_-Nb composite, the NbH_0.6_ phase (solid solution of a metallic hydride) was formed, and the niobium atoms created a structure with vacancies, thereby providing “channels” for hydrogen to flow into the sample.

Kinetic analysis together with practical BM was also conducted by Antisari et al. [93]. Pure MgH_2_ powder was ball milled for 10 h at 0.6 MPa argon pressure. Metallographic observations proved that structural defects enhanced the reaction of Mg with H_2_. Although the authors stated that the MgH_2_ particle density increased with the density of structural defects caused by BM, this result is quite unlikely even from a physical point of view (unless the defects are only interstitial atoms, which is usually not the case). The same research studied the influence of a Fe additive on the hydride, which resulted in a further increase in particle density. Thus, defects were proven to behave like nucleation sites and speed up the phase transformation rate. The paper showed the role of a catalyst on hydrogen absorption and desorption kinetics and suggested that surface catalysis can speed up hydrogen absorption and desorption.

Likely inspired by Reilly and Wiswall [31], whose research suggested the possibility of using Mg_2_Ni as a catalyst for MgH_2_ formation, Huot et al. [82] performed MgH_2_ synthesis with a nickel catalyst. Both hydrogen and argon atmospheres were used during the experiment (the RBM process in that paper is broadly described in the following chapters). When milling a 2Mg + Ni mixture in an argon atmosphere, the presence of intermetallic Mg_2_Ni was noted. This phase had an influence on the decomposition temperature (which increased by approximately 40 °C) and on the decomposition rate. The abovementioned examples should only suggest to the reader the possible effects of the tested metal additives in terms of improving the kinetics of MgH_2_ synthesis and decomposition. However, it must be noted that almost all existing metallic metals have been tried in this context, and describing those trials is far beyond the scope of this work.

#### 2.2.3. Intermetallic Additives

A high hydrogen sorption capacity, low desorption temperature, and better kinetics are general effects of the addition of intermetallic compounds, mainly in La-Ni, ZrNi, ZrMn, and Mg-Ni systems [109,110,111,112,113,114]. Zhou and Ren et al. performed an experiment to examine the effect of Ti and V intermetallic compounds on hydrogen storage properties [115,116]. The lowest desorption temperature was noted for magnesium with added TiMn_2_, but the system had improved absorption kinetics at room temperature while also retaining a high hydrogen storage capacity. A MgH_2_-50% ZrNi compound showed 2.6 wt.% hydrogen desorption at ~275 °C [113]. The authors proposed that a cooperative dehydriding mechanism took place due to elastic interactions at the MgH_2_/ZrMn_2_H_x_ interface.

**Figure 7:**
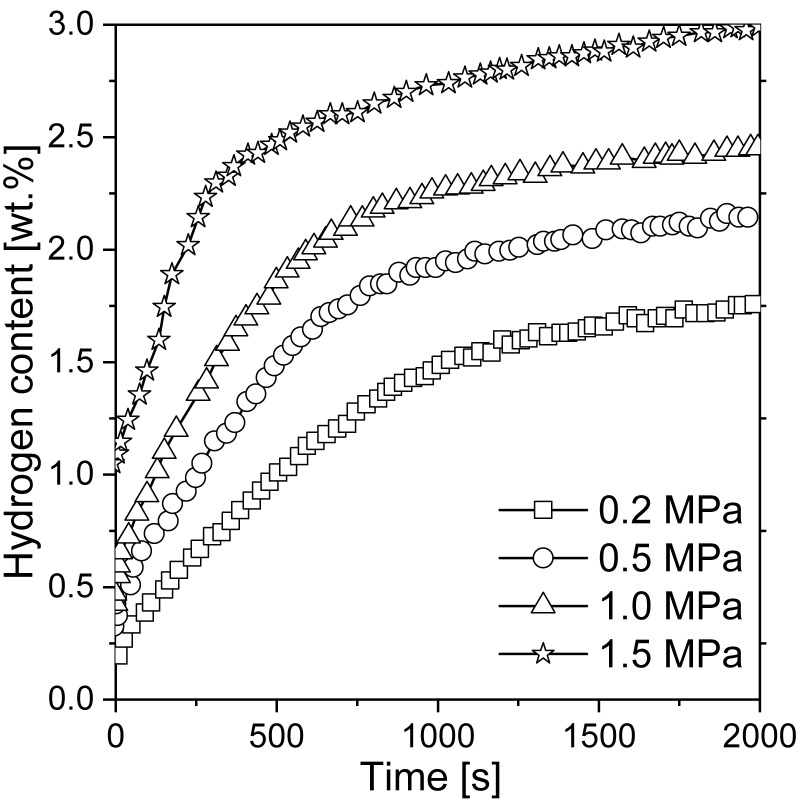
Absorption curves of Mg-50 wt.% LaNi_5_ at 28 °C at different pressures. The graph is based on [117].

Liang et al. [117] mechanically alloyed pure Mg with LaNi_5_ in an argon atmosphere. It was shown that the nanocomposite was not stable during hydrogenation. Thus, it transformed into a mixture of Mg + LaH_x_ + Mg_2_Ni and influenced the kinetics of the reaction. As a consequence, even 4.1 wt.% hydrogen was absorbed at an elevated temperature for 250 s, while 2.5 wt.% hydrogen was absorbed at room temperature for 500 s (with Mg-50 wt.% Ni hydride). Hence, even at room temperature, good absorption kinetics were recorded. The absorption curves of the composite for different pressure values are presented in Figure 7. According to the authors, the fast absorption kinetics at room temperature could be a result of the hydrogen pressure (but only up to 1.5 MPa; nothing changed above that pressure) and temperature. Moreover, the absorption kinetics were highly sensitive to both phases, i.e., Mg_2_Ni and lanthanum hydride, which acted as catalysts for magnesium hydrogenation. The fast diffusion of hydrogen through phase boundaries and nanocrystalline Mg_2_Ni was beneficial to the absorption kinetics. The hydrogen storage properties were influenced by the Mg to Mg_2_Ni and lanthanum hydride ratio. An optimum Mg to Mg_2_Ni and lanthanum hydride ratio was crucial in the context of hydrogen storage properties. Therefore, it was proven that ternary Mg-Ni-La alloys had better sorption kinetics than Mg-La and Mg-Ni binary alloys [118]. Lanthanum hydride had a poor effect on hydrogen desorption but significantly improved absorption. Other studies [119] were carried out with compounds produced by the mechanical milling of La_2_Mg_17_ together with LaNi_5_ as an additive. It was proven that the composite kinetics were improved due to the complex porous agglomeration of the Mg_2_Ni, La, and Mg phases.

Composites of magnesium hydride with AB-type compounds (FeTi in this case) were also studied [120]. It was found that the addition of FeTi lowered the desorption temperature (measured as the position of the DSC peak), and the improvement was correlated with the ratio of intermetallics added.

Based on the literature, it can be said that intermetallics generally improve decomposition kinetics. If intermetallics absorb hydrogen, they can actively influence absorption and desorption. If intermetallics do not have an affinity for hydrogen, they can at least act as grinding agents to improve the mechanical milling process and thus provide a passive influence.

#### 2.2.4. Metal Halide/Oxide Additives

Metal halides and metal oxides influence the hydrogen desorption temperature and the kinetics of both desorption and adsorption of magnesium-based hydrides. Metal oxides can act as agents to refine particles during BM. There is also believed to be an affinity between metal oxides and hydrogen molecules, which enables easy absorption of hydrogen on the surface [21,121,122,123,124,125,126,127].

The catalytic effects of mischmetals and mischmetal oxides on improving the dehydrogenation and rehydrogenation behavior of magnesium hydride (MgH_2_) were reported [128]. Mischmetals (mixtures of rare-earth metals, mostly Ce and La) and their oxides exhibited an influential catalytic effect on improving the hydrogen sorption kinetics and lowering the desorption/absorption temperature of MgH_2_. The best catalyst concentration was approximately 5 wt.% for both catalysts, which mostly affected the hydrogenation kinetics and temperature (in comparison to those of ball-milled MgH_2_ under the same pressure and temperature conditions). The onset desorption temperature decreased by approximately 80 and 60 °C as a result of the catalytic effect of added mischmetal oxide and mischmetal, respectively (compared with that of ball-milled MgH_2_). During the first 10 min, dehydrogenated Mg catalyzed with a mischmetal oxide absorbed 4.75 wt.% hydrogen at 315 °C and 1.5 MPa hydrogen pressure, and the value increased to 5.5 wt.% over 40 min of rehydrogenation. The same conditions were applied to ball-milled Mg and Mg catalyzed with a mischmetal and mischmetal oxide, and as a result, over 40 min, the samples reabsorbed 4.15, 4.62, and 5.43 wt.% H_2_, respectively [128].

**Figure 8:**
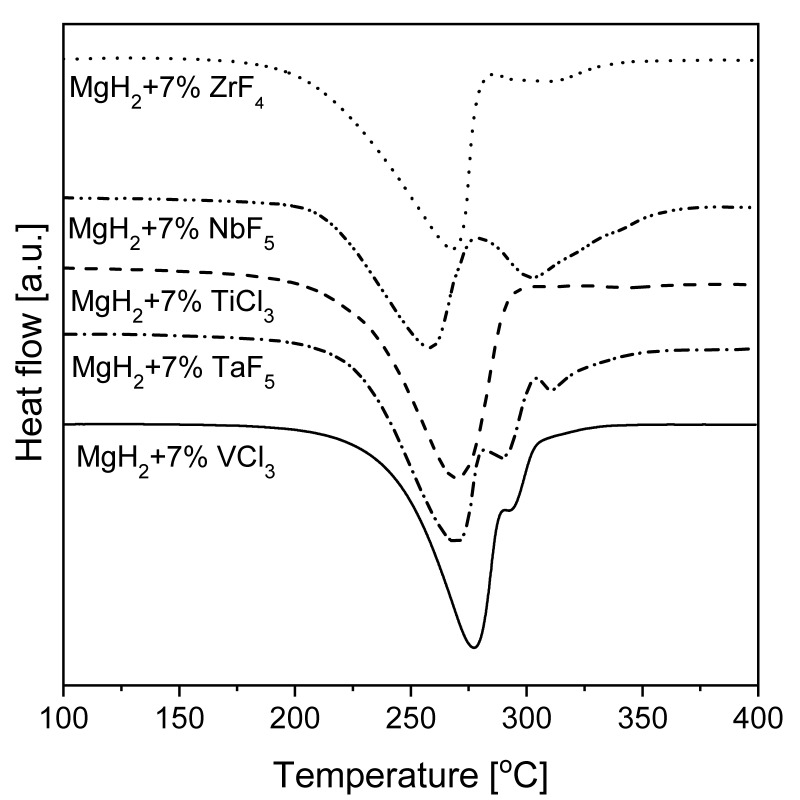
Endothermic peaks of MgH_2_ milled with halides (heating rate 5 °C/min). The graph is based on [131].

The effect of mechanical milling with the use of inorganic salts as magnesium additives on hydriding properties was investigated [129]. The examined halides—NaF, NaCl, MgF_2,_ and CrCl_3_—seemed to have different influences on the surface properties and reaction kinetics. The lowest hydrogen capacity was noted for the Mg-5% MgF_2_ mixture (~4.5 wt.%), while an absorption range of 5.5–6 wt.% H_2_ was achieved for other additives with hydrogenation conditions of 350 °C and 1.5 MPa hydrogen pressure. Thus, it was obvious that MgF_2_ did not act as a catalyst. Additionally, a significant effect on the dehydrogenation kinetics of MgH_2_ was noted. However, the specific surface area increased with an increase in salt content and milling time. Additionally, the influence of various halide additives on magnesium hydride was studied [130]. Some compounds caused a significant change in the hydrogenation kinetics and MgH_2_ decomposition temperature. The strongest catalytic influence was noted for the fluorides NbF_5_ and TiF_3_. All fluorides, except Cu and Y, significantly decreased the decomposition temperature. A possible reason for this behavior was that Cu and Y halides formed stable compounds with Mg which were not active. Nb and Ti fluorides showed the best kinetics improvements, while V, Zr, and Ni showed similar, but slower, kinetics. Fe and Cr fluorides had the least influence on kinetics. The authors claimed that those elements could act in different ways: (a) by forming stable intermetallic phases with Mg but no hydrides and (b) by having a single valency with virtually no catalytic effect. These results meant that the improvement in hydriding properties stemmed not from the fluorides, but rather from the hydrides. The mechanism by which halides increased MgH_2_ decomposition kinetics was studied by Malka et al. [131]. A much stronger influence on the decomposition behavior of MgH_2_ was noted with fluorides than with chlorides (with the exception of TiCl_3_, which decreased the desorption temperature far below 300 °C). The DSC results from this research are shown in Figure 8. The lowest decomposition temperature was 250 °C for the additive ZrF_4_. Many studies have focused on the role of NbF_5_ in the desorption kinetics of ball-milled MgH_2_ [132,133,134]. For noncatalyzed MgH_2_, BM usually changes the decomposition temperature by approximately 30 °C, while with halide addition, a decrease in decomposition temperature of up to 200 °C can be observed.

The catalytic effect of Nb_2_O_5_ was also examined [135], and this compound was found to be one of the best known catalysts for MgH_2_ decomposition. The absorption kinetics turned out to be nearly independent of the catalyst content. Thus, even the addition of a small amount of oxide played a major role in promoting a fast absorption process. It was found that the ternary solid solution Mg_x_Nb_(1−x)_O was the active material responsible for the good kinetics properties [136]. Full hydrogen absorption and desorption (~7 wt.%) were obtained within 60 and 90 s, respectively, for 0.5 mol% Nb_2_O_5_ at 300 °C [88]. The addition of more than 1 mol% Nb_2_O_5_ did not further accelerate the kinetics. The absorption rate at 250 °C was almost two times higher than that at 300 °C, likely due to the high thermodynamic driving force for absorption at lower temperature and the same hydrogen pressure and/or more effective heat dissipation. The mechanism of Nb_2_O_5_ was deeply investigated by Friedrichs et al. [137,138] who proved that it is very unlikely that the oxide itself acts as a catalyst since it is reduced after short-term heating in the presence of magnesium. Thus, niobium (preferably in the form of nanoparticles) might be responsible for the activity. They also found that one of the possible positive effects is that additives prevent magnesium grain growth by occupying grain boundaries. An improvement in the hydrogen sorption kinetics of magnesium hydride powder when using Nb_2_O_5_ as a catalyst was presented by Hanada et al. [89], and this improvement is described broadly in the section on RBM experiments. The effect obtained by Hanada, however, might be due more to the extremely small sample size they used and the resulting improvement in heat exchange.

#### 2.2.5. MgH_2_ Destabilization

Many studies have focused on improving diffusion rates mostly by reducing the particle or crystallite size, which leads to the shortening of diffusion distances and the introduction of defects. However, the thermodynamics of the interaction of hydrogen with magnesium (equilibrium pressure) remain virtually unchanged and clearly will not change due to certain physical reasons. The main reason is the reaction enthalpy, which cannot be simply changed. To tune the desorption thermodynamics of MgH_2_, an intermediate reaction with different thermodynamics must be used. The first pioneering work was performed by Reilly and Wiswall [31], who described the reaction between a Mg-Cu alloy and hydrogen under high temperature and pressure. An improvement in hydrogenation/dehydrogenation thermodynamics was also achieved by using various additive elements to form alloys or compounds with Mg in the hydrogenated or dehydrogenated (or both) states. Zaluska et al. [139] proved that a Mg-Al system had specific properties that allowed a very fast solid-state reaction during the release of hydrogen from MgH_2_. Furthermore, the Mg-Al system had a relatively high hydrogen capacity of up to 3.5–4.5 wt.%. Aluminum improved the heat transfer, thus modifying the hydrogenation thermodynamics. All the abovementioned factors allowed the working temperature of the hydride to decrease. It was proven that the equilibrium pressures for hydride formation could be shifted to a higher pressure range, which made the whole system more stable at low temperatures. Vajo et al. [140] investigated whether magnesium hydrides could be effectively destabilized with silicon. During dehydrogenation of the MgH_2_/Si system, a Mg_2_Si phase formed and caused the equilibrium pressure at 300 °C to increase from 0.18 to more than 0.75 MPa. Additionally, equilibrium pressures of 0.1 and 10 MPa were noted at 20 °C and 150 °C, respectively. However, the kinetics at 150 °C were too slow for direct hydrogenation, which was problematic in terms of using the investigated material for practical hydrogen storage. Later, Bystrzycki et al. [24,25] achieved the destabilization of MgH_2_ by adding silicon and performing a nanoscale solid-state reaction under vacuum. Moreover, Mg_2_Si, which formed as a product of MgH_2_ destabilization by Si, exhibited no hydrogen desorption at temperatures lower than 200 °C. Only the very slow destabilization of MgH_2_ was observed indirectly by pressure changes during desorption at 250 °C after BM of the MgH_2_-Si mixture for 20 h.

#### 2.2.6. Cyclic Stability

Hydride stability upon cycling and its thermal stability during use are important factors in the context of technical applications, mainly due to economic issues (the number of cycles that storage containers can experience without replacing the absorber). The first attempt to examine the cyclic stability of magnesium powder was conducted by Pedersen et al. [141]. Some hydrogen storage capacity loss was observed above 500 cycles. As the cycle number increased, the reaction kinetics decreased. Moreover, while the maximum desorption time turned out to be virtually independent of cycle number, the maximum absorption time was found to be dependent on this factor. More research using the above approach was conducted by Bogdanovic [142,143] and Friedlmeier [144].

**Figure 9:**
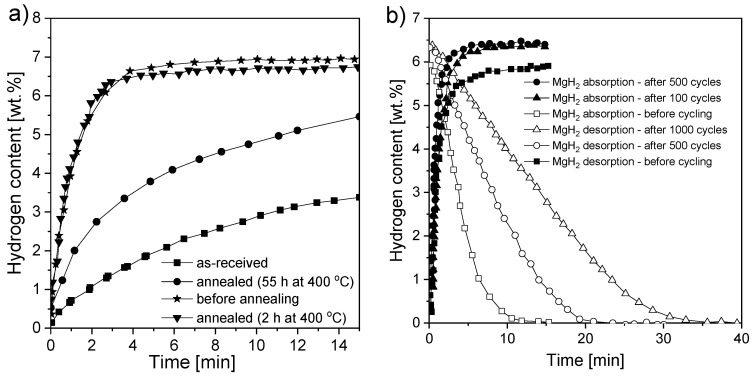
Absorption kinetics of (**a**) pure MgH_2_ (at 350 °C) before and after annealing and (**b**) MgH_2_-0.2 mol% Cr_2_O_3_ after different cycling times. The graphs are based on [126].

Dehouche et al. [126] synthesized MgH_2_ with 0.2 mol% Cr_2_O_3_ to investigate long-term cycling stability and thermal stability. The presence of an oxide catalyst resulted in no need for sample activation. The changes in the thermodynamic and kinetic properties of MgH_2_ in a ball-milled sample were studied and compared with those of annealed and as-received samples. The absorption and desorption properties were tested at 300 °C for 1000 cycles and at 350 °C for 17 cycles. The results are presented in Figure 9a,b. The kinetics changed significantly after the long annealing process but did not change much after cycling. Prolonged annealing decreased the reaction kinetics, which was similar to the results observed for the as-received sample. However, it was noted that the hydrogen storage capacity increased during cycling. Between the 1st and 500th cycle, the capacity increased by approximately 8% (from 5.9 to 6.4 wt.% H_2_). Unfortunately, that improvement was lost after 1000 cycles. The authors concluded that sample cycling had an influence on the desorption behavior. However, the absorption characteristics were found not to be related to the cycle number. This result was opposite to the observation of Pedersen [141], who demonstrated excellent thermal and cycling stability at 300 °C for 1000 cycles. A temperature of 300 °C is sufficient for technical applications (with an equilibrium pressure significantly over 0.1 MPa). These results were in good agreement with a previous investigation of a MgH_2_-5 at.% V composite [125]. Testing the material for 2000 cycles at 300 °C resulted in no significant change in the thermodynamic and kinetic properties. Notably, the hydrogen capacity of the material increased marginally, while a constant rate of absorption and only a small decrease in the desorption rate were observed. Polanski et al. [145] also investigated the effect of Cr_2_O_3_ on the cyclic hydrogen storage behavior of magnesium hydride. After 150 cycles of desorption/absorption at 325 °C, a gradual loss of hydrogen storage capacity from ~5.2 wt.% (after one cycle) to ~4.6 wt.% was observed at the end of cycling. The temperature-programmed desorption (TPD) spectra for hydrogen desorption showed that the decomposition temperature shifted to a higher range as the number of cycles increased. A partial reduction of chromium oxide was observed with the formation of magnesium oxide, as well as crystalline growth in the structure. In general, it may be concluded that the cyclic stability of magnesium hydrides depends on the additives and possible reactions taking place, as well as the hydrogen purity and the way the investigation is performed. Cycling in a “closed-loop” system that uses the same hydrogen usually shows better results than using “fresh” hydrogen each time since even a small amount of impurities may cause powder bed degradation after thousands of cycles.

#### 2.2.7. Carbon Additives

Among the many approaches applied to improve hydrogen sorption/desorption kinetics, BM with carbon additives has been widely examined. To date, magnesium-carbon composites have been synthesized by BM of Mg or MgH_2_ with different carbon allotropes (graphite [146,147,148,149,150,151,152]; nanotubes [150,151,153,154,155]; nanowires, fullerenes and activated carbon [150,151]; amorphous carbon, black carbon and nanodiamonds [150,156,157,158]) and are suggested to be a solution for solid-state hydrogen storage problems. In the past few years, carbon materials have been proven to improve the thermodynamics and kinetics of hydrogen sorption in different hydride systems. One of the most impressive examples of the influence of carbon on metal hydride was shown by Baldé et al. [159], and C-containing additives have been broadly investigated since then.

The main research papers are broadly described in other reviews by Aldelhelm et al. [160], Sun et al. [161], and others [161,162,163,164]. Carbon materials were shown to be suitable in combination with metal hydrides due to their relative chemical inertness. However, possible reactions such as interactions with defects or terminating groups, intercalation, or carbide formation may occur. Moreover, carbon can conduct heat efficiently and can enable hydrogen to diffuse along with the carbon phases, which is beneficial to the sorption properties [160].

One of the first authors investigating the potential of carbon additives with ball-milled magnesium hydride was Imamura et al. [146,147,165,166,167]. His first synthesis was based on mechanical milling of Mg + 5 wt.% Pd-supporting graphite (5 wt.% Pd/G) in the presence of tetrahydrofuran (THF) [146]. The compound absorbed hydrogen even at low pressure (0.06 MPa) and temperature (26 and 180 °C). The samples had very good reversibility for cycling, and the hydriding kinetics were improved with repeated cycles. THF, benzene, or cyclohexane (CH) was used due to the synergic interactions these compounds promote between magnesium and atoms of graphite, which improved the synthesis outcome [147]. Later, the authors characterized mechanically ground Mg-graphite (Mg/G) composites and investigated the influence of different additives (CH and THF) on the compounds [165]. Organic additives strongly affected the physicochemical properties (structures of the surface and the interface) of Mg/G composites and their hydriding characteristics. Magnesium graphite composites with CH and THF are described as (Mg/G)CH and (Mg/G)THF, respectively. CH and THF addition influenced the specific surface area, but as the addition amount increased, the reduction in crystal size stopped. Depending on the process parameters and the type and amount of organic additive, the Mg crystallite size was in the range of 15–26 nm. Moreover, the graphite layer structure in composites with CH and THF was shattered during grinding. Other research aimed to examine the influence of benzene [166].

**Figure 10:**
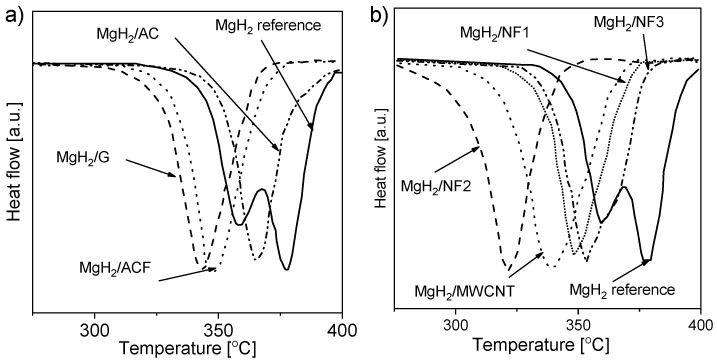
DSC decomposition curves for MgH_2_-carbon compounds containing (**a**) 5 wt.% “conventional” carbons or (**b**) 5 wt.% nanocarbon; both samples were treated by BM for 2 h. The graph is based on [151].

Studies conducted with the BM of magnesium or magnesium hydride showed that the DSC decomposition peak shifted toward lower temperatures (from 380 °C to 300 °C [168] and from 434 °C to ~390 °C [169]). Additional research [151] examined the effect of carbon additives on the hydrogen desorption properties of ball-milled magnesium hydride. The authors investigated the influence of the added “novel” and “conventional” carbons on the alloy. The DSC decomposition curves for both types of carbon are presented in Figure 10 [151]. The decomposition peak shifted by more than 50 °C in some cases compared to that of the reference material. Shang et al. [149] showed that the absorption process was enhanced by adding graphite (1, 10, and 30 mol%) before milling. According to the authors’ statement, the beneficial effect of graphite is connected with preventing oxide film formation on the Mg surface. Nevertheless, the stability of magnesium hydride is still a challenge, and the role of the C-containing compounds remains unclear. For these reasons, more research was conducted to understand the role of novel forms of carbon (carbon nanotubes, nanofibers, fullerenes, graphene, etc.) [151,153,155,158,170,171]. As a result of this research, it was found that the hydriding conditions can be modified by carbon additives (especially when they contain catalytic metal nanoparticles). Nanofibers and multiwalled carbon nanotubes (MWCNTs) alloyed with nickel or iron showed better dehydriding kinetics properties than did other materials.

**Figure 11:**
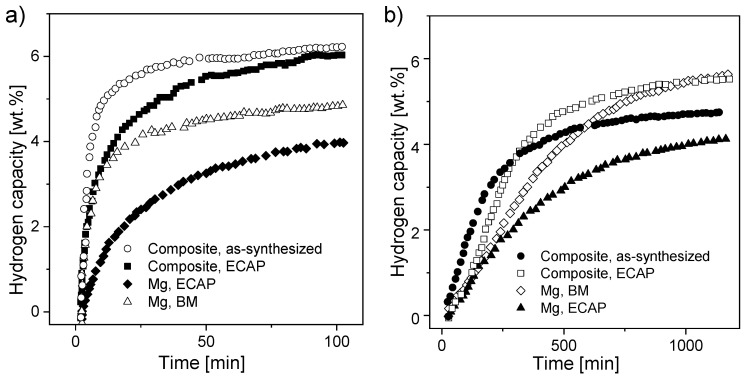
Kinetics of hydrogen (**a**) absorption and (**b**) desorption at 300 °C by as-synthesized and ECAP-processed Mg-2 wt.% MWCNTs compounds compared with 4 h ball-milled Mg and ECAP-processed bulk material. The graphs are based on [153].

Another approach was presented by Skripnyuk et al. [153]. Mg powder and MWCNTs (prepared by the decomposition of acetylene with the aid of 5% Co, Fe/CaCo_3_) were milled for 4 h and then hot pressed under a pressure of 50 MPa at 600 °C. The hydrogen absorption/desorption kinetics at 300 °C of Mg-2 wt.% MWCNTs was found to be much faster than that of reference samples of pure magnesium processed by BM or equal-channel angular pressing (ECAP). The as-synthesized composite could reversibly store approximately 7 wt.% hydrogen (Figure 11a,b) and exhibited increased equilibrium hydrogen pressures of adsorption and desorption at high hydrogen contents. The authors suggested that the improved kinetics were connected with the fast diffusion of hydrogen through the MWCNT cores, and they proposed that the increase in the equilibrium pressure of hydrogen is related to the elastic constraints placed on the magnesium matrix by the carbon nanotubes. Other exceptional research was provided by Sartori et al. [172]. In addition to adding C-containing compounds to the magnesium alloy, a metal oxide (Nb_2_O_5_) was mixed with the material. The absorption kinetics of the Mg-20 wt.% MWCNT composite worsened, and the absorption rate slowed. With added Nb_2_O_5_, the enthalpy of hydride formation was almost unaffected, so it can be concluded that neither graphite nor niobium oxide influenced the absorption/desorption thermodynamics.

### 2.3. Reactive Ball Milling

Another approach to MgH_2_ synthesis involves milling in a hydrogen atmosphere. Mechanochemical activation improves hydrogen diffusion into the material by lowering the diffusion distance, which is a significant feature of the hydriding reaction. BM of alloys in a hydrogen atmosphere causes both hydrogen uptake and mechanical deformations to occur simultaneously. RBM, due to the high mechanical pressures, promotes the formation of high-pressure polymorphs of powders. Thus, the metastable γ phase is visible [53]. The first attempt at a reactive synthesis of MgH_2_ was reported by Chen and Wiliams [83]. The experiment showed the possibility of fast metal hydride synthesis by mechanical alloying (MA) from metal powders (magnesium, zirconium, or titanium) in a relatively low-hydrogen-pressure atmosphere (0.1 MPa) at room temperature. It was proven that the properties of the obtained products (in terms of decomposition kinetics) were even better than those of materials produced by conventional methods. The hydriding process occurred as a two-step reaction, which ended when a stable pressure level was achieved. During the investigation of the product, an endothermic reaction was noticed in the DSC graph at 382 °C, and the total weight loss for the Mg sample was 5.60 wt.% (from the TGA data). Additionally, the measured hydrogen content (established by combustion elemental analysis) was 7.46 wt.%. According to the authors, this inconsistency in results was connected with the occurrence of oxidation during heating. The above sample showed an almost 2% absolute (>20% relative) difference when measured by two different methods, thus prompting the reader to consider which result is more accurate.

#### 2.3.1. Metal/Semimetal Additives

It is well known that introducing even a small amount of metal additives into magnesium can significantly improve the hydrogen absorption and desorption kinetics. The most popular metal catalysts are transition metals, e.g., Fe, Pd, Ni, V, Nb, Ti, Mn, and Cr (synthesized together with Mg by BM and RBM techniques) [85,94,95,96,97,98,173]. Ryoung et al. [106] investigated the influence of Mg, Ni, Fe, and Ti additives on the hydriding and dehydriding properties of Mg. Long milling times led to low initial sorption rates and low amounts of absorbed and desorbed hydrogen. Reilly and Wiswall [31] noted the possibility of using Mg_2_Ni as a catalyst for MgH_2_ formation. With this knowledge, an attempt to synthesize MgH_2_ by reactive milling was conducted by Huot et al. [82]. Notably, pure nickel does not absorb any hydrogen in a reasonable temperature and pressure range [174]. The MgH_2_ decomposition DSC peak for pure Mg that was milled for 25 h in H_2_ was observed at 440.7 °C (10 °C/min heating rate). Even a small addition of nickel significantly decreased this temperature to 225.4 °C. Neither Mg_2_Ni nor the Mg_2_NiH_4_ phase was observed, in contrast to the results for BM in an argon atmosphere.

A broad analysis of the Mg-Ti, Mg-V, and Mg-Nb systems in terms of the most effective additive for improving hydrogen interaction properties was performed by Korablov et al. [97]. Hydrogen uptake, especially at room temperature, was the highest with the vanadium additive (demonstrating the highest degree of conversion into the hydride phase). On the other hand, the titanium additive resulted in the lowest activation energy, which was beneficial for the dehydrogenation process. De Castro et al. [175] performed a synthesis of MgH_2_-5% Nb and determined cooperative behavior at the Mg/Nb nanointerface and its catalytic effect on the reaction kinetics. The authors showed that Mg promoted Nb hydrogenation as much as Nb promoted Mg hydrogenation. The niobium additive improved the hydrogenation process, and NbH_2_ was observed. The influence of Pd and Ni additives on magnesium hydride synthesized by RBM proved that the combination of these two elements provided great cyclic stability and improved kinetics under moderate absorption conditions [176]. Another important study [177] proved that different particle sizes of catalytic additives (Co in that case) and different types of lattices had different catalytic effects on MgH_2_ (different hydrogen storage capacities and different sorption kinetics). Kral et al. [94] improved hydrogen storage properties by utilizing the catalytic effects of Mg-Al-Ti-Zr-C powders. The synergistic effect of the phases caused increased hydrogen desorption enthalpy, with a value that was close to the lowest published values for MgH_2_.

The hydrogen desorption properties of a catalyzed MgH_2_ composite prepared by mechanical milling of a mixture of MgH_2_ with transition metal nanoparticles were measured by Hanada et al. [178]. Iron, cobalt, nickel, and copper particles were used as catalysts in RBM (in a 1 MPa hydrogen atmosphere). The nanocrystalline particles could be activated much more easily than their polycrystalline analogs. All synthesized composites showed improved H_2_ desorption properties. However, Ni nanoparticles had the largest influence on the hydrogen desorption properties. A mixture of MgH_2_ with 2 mol% Ni had an ~6.5 wt.% hydrogen capacity and was characterized by a peak temperature that was ~100 °C lower than that of MgH_2_ alone. However, a small decrease was noticed during the second cycle. The authors suggested that this decrease occurred because a Mg_2_Ni phase formed at the boundary between MgH_2_ and Ni after hydrogen desorption, thereby forming a ternary hydride with low capacity. No simple correlation between the particle and crystallite sizes and the desorption kinetics was noted. Long milling (>15 min) led to better kinetics by increasing the activated surface area. Hence, the increased activated surface area decreased the hydrogen desorption activation energy. All of these factors resulted in 90% hydrogen desorption at 163 °C in He gas flow without a partial hydrogen pressure during the first 100 min of the process. The improvement in the hydrogen desorption kinetics was said to be strongly correlated with the change in the microstructure, i.e., the formation of a composite material.

**Figure 12:**
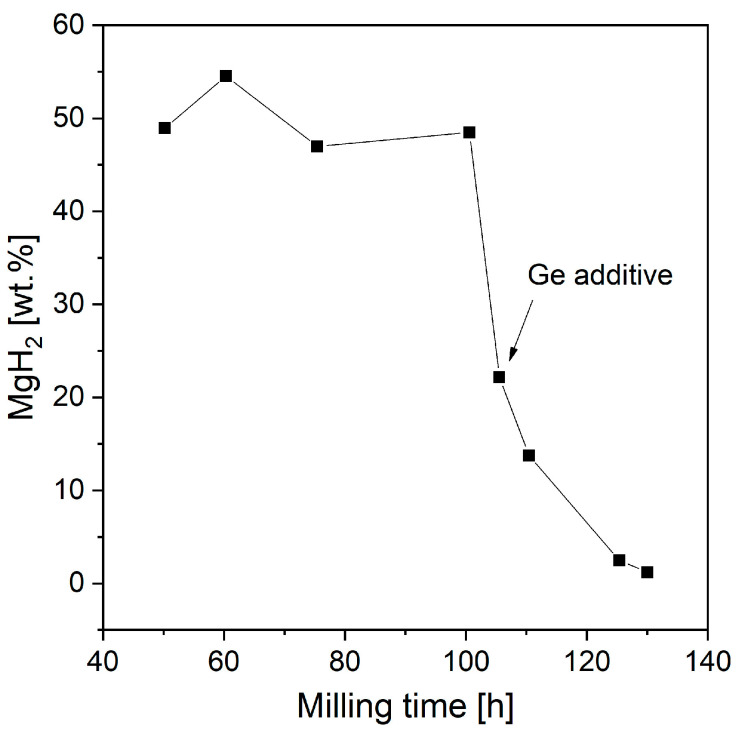
MgH_2_ yield as a function of milling time for samples with and without added Ge. The graph is based on [179].

Gennari et al. [179] used RBM to produce a mixture of Mg and Ge. Ge addition led to significant structural modifications and changes in desorption properties. Studies have proven that with increased milling time, the MgH_2_ phase will destabilize and Mg_2_Ge will form. Figure 12 shows that after 100 h of milling with added Ge, the amount of magnesium hydride started to decrease (based on DSC and XRD measurements). Moreover, milling Mg with Ge led to a low MgH_2_ decomposition temperature (compared with that of samples produced by BM of only Mg). The overall catalytic effect was noted to be independent of Mg_2_Ge but strongly dependent on Ge. The authors deduced that Ge could provide special areas for hydrogen atom transfer from the bulk to the surface, which favored the recombination of molecular hydrogen. Another hypothesis was that Ge could generate alternative diffusion paths, thus improving hydrogen mobility through the material. However, the mechanism itself has only been proposed and has not been proven.

**Figure 13:**
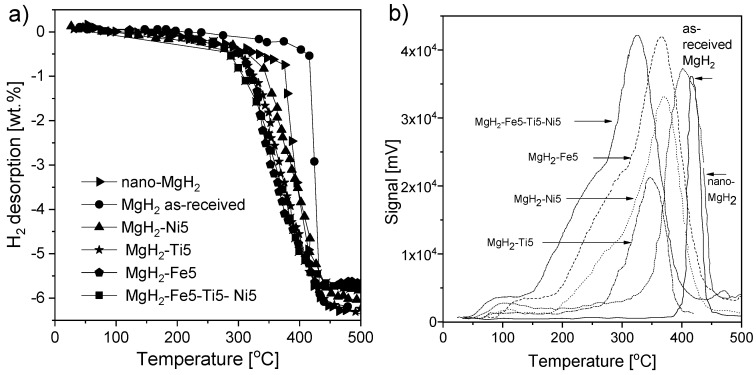
(**a**) Temperature dependence of the hydrogen content of MgH_2_ with and without catalysts. (**b**) TPD (peak at maximum desorption) of MgH_2_ with and without a catalyst. The graphs are based on [180].

Sashi et al. [180] provided the results of MgH_2_ synthesis with the most catalytically effective transition metals, i.e., Ti, Fe, and Ni, and the influence of each catalyst on the sorption characteristics was investigated. Magnesium hydride milled together with 5 wt.% of each element in a 1.2 MPa hydrogen atmosphere led to the formation of nano-Ti5Fe5Ni5, nano-Ti5, nano-Fe5, and nano-Ni5. The decomposition temperature of the produced materials was lower than that of nano-MgH_2_ alone, while the rehydrogenation kinetics were enhanced due to the cocatalyst effect of Ti, Fe, and Ni. The hydrogen absorption value for nano-Ti5Fe5Ni5 was 5.3 wt.% at 270 °C and 1.2 MPa H_2_ pressure during a 15 min cycle, while a reabsorption value of 4.2 wt.% was obtained for nano-MgH_2_ under identical conditions (Figure 13a). The desorption temperature, with a visible peak at the maximum desorption rate, of different samples is presented in Figure 13b. For MgH_2_-Ti5Fe5Ni5, the decomposition temperature was 280 °C, which was the lowest temperature obtained for the examined samples (310, 320, 340, and 370 °C for nano-Fe5, nano-Ti5, nano-Ni5, and nano-MgH_2_, respectively).

#### 2.3.2. Oxide Additives in Reactive Ball Milling

**Figure 14:**
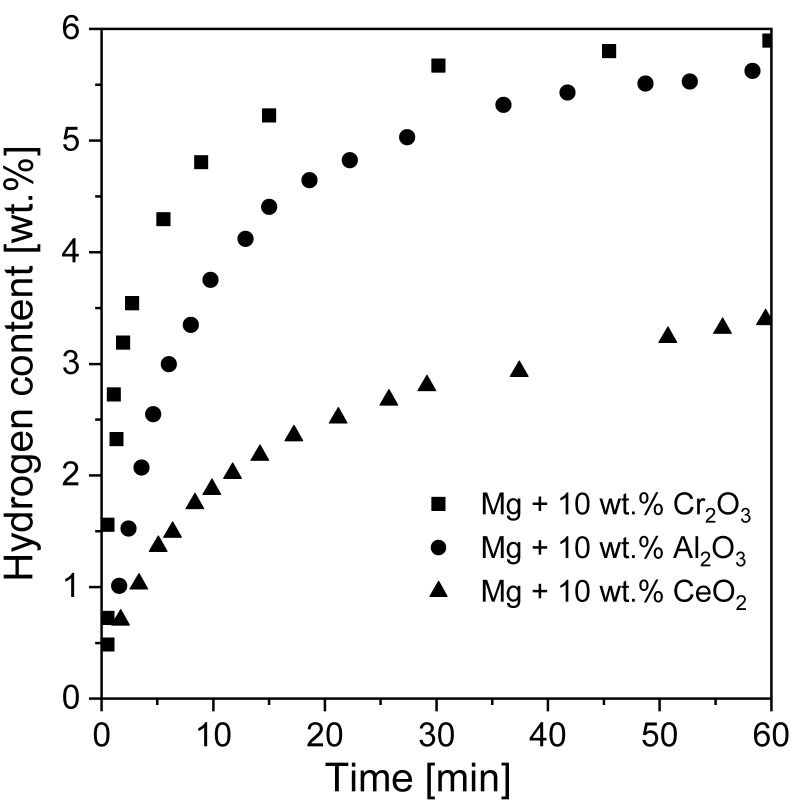
Weight percentage of H_2_ absorbed over time for the first hydriding cycle at 300 °C at 1.1 MPa H_2_. The graph is based on [182].

Crucial changes in hydriding behavior have been noted when using metal oxides as catalysts [21,121,126,181]. Song et al. [182] showed that the oxides Cr_2_O_3_, Al_2_O_3_, and CeO_2_ influenced magnesium hydride during RBM. After 2 h of RBM, the Mg was only partially transformed into MgH_2_ (20.6, 10.1 and 13.7 wt.% MgH_2_ from the Mg + 10 wt.% Cr_2_O_3_, Mg + 10 wt.% Al_2_O_3_ and Mg + 10 wt.% CeO_2_ phases, respectively). The largest hydriding rates of absorption and desorption was noted for Mg + 10 wt.% Cr_2_O_3_ powder. It absorbed 5.87 wt.% at 300 °C in a 1.1 MPa H_2_ atmosphere and desorbed 4.44 wt.% at 300 °C and 0.05 MPa H_2_ over 60 min in the first cycle. Absorption curves for all powders are presented in Figure 14. The amount of absorbed/desorbed hydrogen increased with decreasing particle size because the diffusion distance became shorter. In the case of Cr_2_O_3_, hydriding/dehydriding cycles led to its reduction because of the much greater chemical affinity of magnesium to oxygen than chromium. In fact, many transition metal oxides become reduced when in contact with magnesium. However, most of the published data about these added transition metal oxides do not deeply discuss how the oxides transform. Instead, the initial states of the oxides (as well as halides) are considered input variables when discussing these catalysts.

Another study showed results for BM of Mg-10 wt.% Fe_2_O_3_ under H_2_ pressure [183]. Defects created on the surface of Mg particles with decreased size (easier nucleation) showed increased hydrogen absorption, up to 5.56 wt.%. It was shown that the absorption rate changed with milling time because of the reduced particle size of Mg, which decreased the diffusion distances. The reaction kinetics improved with milling time since, as already mentioned, materials with nanocrystalline particles are characterized by a low packing density. This low packing density resulted in faster diffusion through grain boundaries than through the less distorted lattice of a microcrystalline material. Grain boundaries acted as nucleation sites for hydride phase formation and decomposition. Another improvement in the hydrogen sorption kinetics of magnesium hydride powder was accomplished with the use of Nb_2_O_5_ as a catalyst, as presented by Hanada et al. [89]. Kinetics curves of the hydrogen absorption and desorption reactions for powders ball milled for 20 h showed up to 5 wt.% hydrogen absorption at room temperature at 1 MPa (after dehydrogenation at 200 °C). Even at room temperature, the product quickly absorbed hydrogen at pressures lower than 0.1 MPa. After rehydrogenation, the material desorbed ~6 wt.% hydrogen at 160 °C for 100 min. The desorption reaction was conducted in a helium flow with a zero partial pressure of hydrogen (otherwise, it would be impossible to desorb hydrogen at this temperature). Thus, Nb_2_O_5_ was proven to cause a sufficient decrease in the activation energy of hydrogen desorption.

#### 2.3.3. Polymorphic Forms after Synthesis

RBM is a synthesis technique in which the existing phases of the examined materials play a crucial role. Knowledge of these phases is necessary to understand the structural changes that can influence the synthesis outcome. An analysis of the existence of β and γ phases after RBM was performed by Gennari et al. [184]. Thermal behavior examination proved the influence of milling time on the properties of the formed hydrides. Three types of desorption behavior were noticed to be connected with the structural changes introduced by RBM. The phase content for each sample was dependent on milling time. With shorter milling times, only the β–MgH_2_ phase was observed as a sharp endothermic desorption peak. Both phases were visible after milling for a long time, appearing as two peaks or as a double peak of the endothermic reaction. The γ-MgH_2_ phase decomposed before the transformation from γ to β. The authors deduced that there was a synergistic effect between the two phases during hydrogen desorption, which caused the β–MgH_2_ desorption temperature to decrease. As a result, the hydrogen desorption properties were influenced by increases in milling time. MgH_2_ synthesis and reaction analysis were also presented by El-Eskandarany et al. [185]. The authors claimed that under RBM in a hydrogen atmosphere, cyclic phase transformation between the two phases β and γ took place with increasing milling time. Therefore, it was suggested that the formation enthalpy values for both phases were similar, with a relatively low energy barrier between them, thereby allowing for cyclic phase transitions. Long RBM times led to a decrease in the grain size of MgH_2_ and a simultaneous increase in Fe contamination level. Iron contamination was introduced by using steel milling tools. Both factors improved the kinetics of absorption/desorption. For powders ball-milled for 200 h, a high hydrogen storage capacity (7.54 wt.%) was noted after the completion of 600 absorption/desorption cycles at 300 °C. In general, this kinetics improvement was achieved because of the nanocrystalline nature of the powders, the presence of the γ phase and Fe contamination. However, the above results seem to be very controversial, and most likely, the described phenomena (cyclic transformation from β to γ) should be analyzed again to assess their validity. Both γ-MgH_2_ and nanostructured MgH_2_ were also synthesized by a direct reaction in a ball mill in a hydrogen atmosphere with the catalytic effect of added ZrFe_1.4_Cr_0.6_ [186]. The formation of the γ-MgH_2_ phase was achieved by applying proper conditions, in which high-energy collisions were conducted with steel balls.

#### 2.3.4. Carbon Additives in Reactive Ball Milling

One of the most popular carbon additives is graphite, which can be milled with magnesium hydride under a hydrogen atmosphere [154,157,187,188]. Fuster et al. [189] performed a broad investigation of the role of carbon additives, noting some aspects of the influence of carbon on the hydride and some inconsistencies. For example, some authors claim that graphite acts as a catalyst to improve hydrogen sorption [157,188], while others disagree [151,190]. Fuster et al. [189] proved that the addition of graphite improves the hydriding rate by reducing the time required to achieve a full reaction with hydrogen by half. Moreover, graphite restrained the cold welding of Mg particles, which caused particle size refinement and thus a more efficient hydriding process. However, the catalytic effect of graphite seemed to be relevant on the surface of Mg particles, which was attributed to the increased decomposition temperature with milling time. Given the characteristics of graphite and its lubricating properties, a possible explanation might be that the mechanical, not chemical, properties of the additive play a role here. A similar phenomenon was previously observed when steel (instead of iron) was used as the synthesis substrate in the formation of ternary hydrides. Despite the very similar compositions, differences in the mechanical properties and conditions of BM were found to be responsible for the final synthesis results and properties of the obtained material [49]. This deviation is an important issue and hints that not only the chemical composition should always be considered when discussing the influence of additives.

Other research was performed by Chen et al. [154]. In addition to adding C-containing compounds to a magnesium alloy, some metals were also mixed in. Composites of magnesium, MWCNTs, and suitable additives (zirconium for improving the absorption kinetics and nickel as a binder) were prepared by RBM to obtain a better hydrogen source for proton exchange membrane (PEM) fuel cells [154]. The obtained microstructure was on the nanometer scale (several to several dozen nanometers, as proven by XRD and transmission electron microscopy (TEM) analysis). The maximum hydrogen storage capacities of Mg-5 wt.% MWCNTs and Mg-20 wt.% MWCNTs were 6.08 and 2.75 wt.%, respectively, at 280 °C. The Mg-5 wt.% MWCNTs composite retained its maximum capacity and exhibited a good reaction kinetics rate under certain temperature and pressure conditions. Huot et al. [187] showed faster decomposition kinetics at 350 °C due to the presence of graphite. Huang et al. [191] showed that the desorption temperature of the rehydrogenated compound decreased by approximately 35 °C (in the range of 400 °C) in comparison to that of the as-prepared compound.

### 2.4. Nanoconfinement

Among the abovementioned methods of modifying magnesium-based hydrides, nanoconfinement is also important. Nanoconfinement is, in general, a modification method based on two aspects: nanosizing and confinement. In theory, nanoconfinement is supposed to significantly enhance kinetics and modify thermodynamic properties by lowering the size of clusters to the extent that the hydride becomes less stable. Accordingly, three possible mechanisms can be distinguished: the first option is reducing the particle size of the hydride, the second option is hindering the growth and agglomeration of particles by compartmentalizing the nanoparticles with the use of a scaffold material, and the third option is limiting the mobility of the decomposition products and preserving the distance between them. Nielsen et al. [68] published a broad review regarding the utilization of nanoporous materials as scaffolds to prepare and confine nanosized metal hydrides. The review aimed to highlight important aspects of nanoconfined chemistry for hydrogen storage materials in the context of hydride characteristics such as kinetics, stability, and thermodynamic properties. The authors mainly focused on the preparation and properties of light metal hydride nanocomposites infiltrated into nanoporous scaffold materials [68]. Another broad review article regarding modification through nanoconfinement was provided by Zhang et al. [56,164]. The authors focused on different ways of modifying one of the most promising candidates for hydrogen storage—magnesium hydride. In addition to describing the influence of C-containing compounds (1D, 2D, and 3D) on the properties of MgH_2_, the authors also focused on the importance of catalysts in the context of nanoconfinement.

From the synergetic effect of nanoconfinement with catalysts, three working principles can be distinguished in terms of where the nanoconfined materials are confined: (a) in both Mg/MgH_2_ and the catalyst, (b) in only the catalyst, and (c) in the catalyst, which plays a dual role of nanoconfinement and catalysis [56]. One of the problems with catalyst-added Mg-based composites is a reduction in capacity due to particle agglomeration. Several methods have been proposed for confining Mg/MgH_2_ + catalyst (Pd, ZrO_2_, Nb_2_O_5_, etc.) systems. Many studies have examined the influence of additives such as carbon nanotubes (CNTs), nanorods, graphene, and nanofibers [192,193,194,195,196,197,198,199,200]. All of this research has proven that mixed compounds of magnesium-based hydrides with catalysts and carbons influence the hydride synthesis outcome. Ranjbar [195] proved that 5 wt.% CNTs added to MgH_2_ + 10 wt.% Ti_0.4_Mn_0.22_Cr_0.1_V_0.28_ lowered the initial temperature of desorption by approximately 125 and 59 °C for the pure sample and the binary mixture, respectively. The gravimetric capacities at 300 °C and 250 °C were 6 wt.% and 5.6 wt.% hydrogen, respectively.

The nanoconfinement of magnesium hydride is still not fully known, and more research is needed on this topic. Nevertheless, alloying effects clearly lead to kinetic enhancements and thermodynamic property alterations. Many studies have proven an apparent improvement in the hydrogen sorption properties of nanoconfined materials compared to those of bulk samples [67,201,202]. Nanoconfinement can be considered a milestone in regard to improving the kinetics and thermodynamic properties of hydrides.

## 3. Mg_2_FeH_6_

Mg_2_FeH_6_ is another magnesium-based hydride that has been commonly investigated. The first information about this compound is from 1971, but since it was only in the title of the conference paper [203] that can no longer be accessed, it is difficult to judge now whether the author synthesized the compound or just predicted its existence. Mg_2_FeH_6_ has a cubic K_2_PtCl_6_-type structure [35] (with no known polymorphs) and the highest known volumetric density of hydrogen (150 kg/m^3^) compared with those of all “popular” hydrides. The gravimetric density is 5.43 wt.%, and the most commonly provided desorption enthalpy, calculated from Van’t Hoff’s equation by Bogdanovic [37] and confirmed by Reiser [204], is −77.4 kJ/mol H_2_. This value is different from data reported by Gennari [38] and Wang [205] (values equal to −98 kJ/mol H_2_ and −67 kJ/mol H_2_, respectively). Because the enthalpy value is lower than that of magnesium hydride, Mg_2_FeH_6_ is more stable (Mg_2_FeH_6_ has a lower decomposition pressure than MgH_2_ at the same temperature). On the other hand, the volumetric capacity of Mg_2_FeH_6_ is higher than that of MgH_2_, which seems to be a very large advantage. The synthesis of magnesium-iron hydride has always been described as complicated because of the lack of a Mg_2_Fe intermetallic phase [39]. This feature is in contrast to the properties of Mg_2_NiH_4_, which has a Mg_2_Ni intermetallic precursor. Fe is also not significantly soluble in Mg (in either solid or liquid form), and any intermetallic compound can be formed from both elements [206]. However, given the many successful attempts at Mg_2_FeH_6_ synthesis, it is hard to judge whether using an intermetallic phase as a starting material will give any advantage compared with already known methods. An advantage may be observed in mass production, for instance, when massive ingots can be cast and then hydrogenated. Thus, the above complex hydride was first reported to be successfully synthesized in 1984 by direct hydrogenation–sintering of Mg and Fe powder mixtures [35]. The process was conducted at 2–12 MPa hydrogen pressure for several days at ~500 °C, and the product was formed with a purity of approximately 50% according to reaction (1).

2Mg + Fe + 3H_2_ ⇄ Mg_2_FeH_6_. (1)

Mg_2_FeH_6_ should form as the first product during hydrogenation because it is more stable than magnesium hydride. However, a quite common and effective path is a two-step reaction with MgH_2_ acting as a Mg_2_FeH_6_ precursor [49,207,208,209,210,211]. Usually, hydride synthesis involves sintering at elevated temperatures (up to 500 °C) and at a high hydrogen pressure. Mg_2_FeH_6_, a complex hydride, can also be prepared by MA/milling (MA/M) (RBM under a certain hydrogen pressure or in an inert atmosphere). Both techniques can be combined with sintering, but they can also exist separately. Furthermore, both a mixture of MgH_2_ + Mg_2_FeH_6_ and Mg_2_FeH_6_ alone are appropriate materials for thermal energy storage (unfortunately, at a high temperature of ~500 °C) due to their high cycle life, relatively low cost of production (including raw materials) and constant and significant heat delivery without heat loss [37,212,213,214,215].

### 3.1. Mg_2_FeH_6_ Synthesis/Mechanical Modifications

The conventional method of preparing pure magnesium-iron hydride (Mg_2_FeH_6_) involves sintering at temperatures ranging from 350–500 °C and at high hydrogen pressure (1.1–12 MPa) over several days [35,216]. An improvement to this synthesis method was made by applying MA/M in an inert or hydrogen atmosphere, a process known as reactive mechanical alloying (RMA). The above methods can be combined with sintering techniques [36,214,216], but mostly, MA is used alone to produce the hydride. Bogdanovic et al. [37] carried out thermodynamic and microstructural investigations of Mg_2_FeH_6_, and the initial formation and subsequent de- and rehydrogenation process of Mg_2_FeH_6_ and the mixed Mg_2_FeH_6_-MgH_2_ system at the micro- and nanoscale levels were investigated. Both the reversible Mg_2_FeH_6_ and the Mg_2_FeH_6_-MgH_2_ mixture had high gravimetric and volumetric densities. Both systems had very good cycling stability, and the hydrogen storage capacity of the latter system had a high potential to be improved by varying the cycling conditions. Zhou et al. [217] studied the energy and electronic structure of Mg_2_FeH_6_ by using the first-principles plane-wave pseudopotential method to calculate the heats of formation and the formation mechanism, which was then proven by experimental methods. The mechanism proposed by the authors is as follows: H atoms are first dissolved in the magnesium lattice, forming MgH_2_, and then iron dissolves into the lattice to form a (MgFe)H_2_ solid solution. With increasing Fe concentration, Mg_2_FeH_6_ is formed.

Mg_2_FeH_6_ has been broadly synthesized with various proportions of initial compounds by using different methods that have resulted in different outcomes. The Mg to Fe ratio can be extremely different, ranging from 1:1 to 40:1 [37]. Puszkiel et al. studied compositions from 2:1 to 15:1 [218]. It has also been shown that it is possible to produce a material with a relatively high hydrogen capacity (up to 5.2 wt.% H_2_ after 550–600 cycles) even without BM [37] by just thermal cycling. Magnesium-iron hydride has attracted attention not only for its high volumetric hydrogen density but also as a thermochemical storage system. The material absorbs heat and releases hydrogen during desorption, while the stored heat is released during hydrogenation and can be utilized. Recently, it has been shown that the Mg-Fe-H system can be used for short- and long-term storage applications at temperatures up to 550 °C [219]. Moreover, the electrochemical reaction of lithium ions with Mg_2_FeH_6_ has also been investigated [220]. Previous research [37] proved that Mg_2_FeH_6_ could be formed from elemental Mg, Fe, and H_2_ without MgH_2_ as an intermediate product. Different milling parameters were applied to optimize the process and increase the efficiency of the reaction. There is still little research showing the results of mechanochemical synthesis without further sintering or other techniques. Hence, there are four main methods for obtaining Mg_2_FeH_6_ using MA: (a) milling Mg and Fe in an inert atmosphere (e.g., Ar) and then hydriding the material, (b) milling Mg and Fe in a H_2_ atmosphere (RMA), (c) milling MgH_2_ and Fe in an inert atmosphere, and (d) milling MgH_2_ and Fe in a H_2_ atmosphere.

### 3.2. Ball Milling

Although magnesium and iron are known for their immiscibility in classical metallurgical processes, the first attempt to mechanically alloy Mg and Fe was conducted by Hightower et al. [221]. The main aim of the research was to determine a possible increase in the solid solubility of either Mg in Fe or Fe in Mg. The results showed that Mg and Fe milled together formed very finely dispersed clusters of one phase in another. Alloys with less than 20 at.% Mg were found to have only one phase, namely BCC, with an enlarged lattice parameter. On the other hand, a high Mg concentration (from 20 to 95 at.%) led to a mixture of both bcc and hcp phases. These results strongly suggested the increased solubility of magnesium in iron. However, the authors stated that there was no strong evidence of achieving up to 20% magnesium dissolved in iron.

#### 3.2.1. Reaction Yield Analysis

The success of the hydrogenation process is indicated by the yield of the reaction. An initial problem limiting the effectiveness of Mg_2_FeH_6_ synthesis is based on the presence of Fe, MgH_2_, Mg, and MgO contamination. J. Huot et al. [222] presented an approach for Mg_2_FeH_6_ synthesis by BM without further sintering. After 60 h of milling MgH_2_ with Fe in argon, 55 wt.% Mg_2_FeH_6_ was synthesized. A very fine microstructure was obtained, which was quite the opposite of that obtained by sintering, and this fine microstructure exhibited better reversibility. Additionally, the loss of hydride capacity upon cycling decreased. Notably, a low yield was caused by an obvious hydrogen deficit in the reaction since the milling was conducted in argon. Puszkiel et al. [212] showed hysteresis phenomena during the absorption-desorption cycle. Mechanically milled material in an argon atmosphere was further sintered to obtain 49 wt.% Mg_2_FeH_6_, 18 wt.% MgH_2,_ and 6 wt.% and 27 wt.% unreacted Mg and Fe, respectively. With the formation of Mg_2_FeH_6_ and MgH_2_ in the sample, the observed hysteresis tended to increase in size. The rate of hydrogen uptake increased with increasing temperature (in the range between 300 °C and 350 °C), while the total hydrogen content was 4.5 wt.%.

The influence of milling time on the yield of Mg_2_FeH_6_ was examined for a ball-milled mixture of MgH_2_ and Fe powders in a two-step reaction by Polanski et al. [207,209]. The authors proved that the optimization of milling time was crucial, and the best yield (>94% Mg_2_FeH_6_) was observed after 2 h of milling (so-called mechanical activation) with the setup used. Prolonged milling resulted in low yields after sintering. The two pressure plateaus suggested the occurrence of two phases (MgH_2_ and Mg_2_FeH_6_), which was proven by XRD analysis. The authors could not precisely explain the phenomena behind the higher yield of the sample milled for two hours. However, they provided some hypotheses. Mechanical milling, in addition to forming a composite and lowering the diffusion distance, caused lattice deformation and partial amorphization. Hence, it is crucial to choose proper milling parameters. Later, a synthesis “efficiency map” was provided by Witek et al. [210] for the same two-step synthesis. The dependence of the Mg_2_FeH_6_ yield on the sintering temperature of the powders and the BM time was presented, providing an easy tool for readers to choose processing windows to obtain the best synthesis results. Yield calculations for each synthesis were based on a comparison of the closed crucible mass of samples before and after synthesis. Samples that were sintered at the same temperature had a highly similar phase composition despite differences in milling time. A yield of 97% was recorded for samples sintered at 500 °C, which suggested almost full transformation. These results proved the research findings mentioned above. Too long or too short milling time was not beneficial for hydride synthesis, especially at low temperatures. It was suggested that this dependence might not be connected with the reaction kinetics but with the material properties after milling. A long milling time could lead to distinctly low heat conductivity. For this reason, samples with a high volume sintered for a relatively short time could not fully transform due to the lack of temperature equilibrium over the sample volume. With insufficient time, even further sintering would not guarantee full transformation. The authors suggested that since perfectly pure powders (substrates) cannot be assumed, a 100% yield is clearly not possible, especially given the significant problems associated with obtaining MgH_2_ of high purity (more than 90%) in reality.

Since it is difficult to perform magnesium-iron hydride synthesis with a reaction yield higher than 90%, Nyallang et al. [223] preceded their synthesis by heat treatment of the starting materials (2Mg + Fe elemental powders). A maximum reaction yield of 84% was obtained in less than 5 h of milling. The two-step reaction-based synthesis exhibited very fast reaction kinetics and low thermal stability (in comparison to those of MgH_2_). These results were explained by Fe acting as a catalyst for the MgH_2_, Mg_2_FeH_6_, and Fe mixture.

#### 3.2.2. Formation Mechanism Analysis

The formation mechanism of complex hydrides of Mg_2_FeH_6_ under equilibrium conditions has been broadly investigated recently [38,209,210,224,225]. In most cases, hydride synthesis results in a MgH_2_-Mg_2_FeH_6_ hydride mixture [35,36,37,38,204,226]. The above studies showed two different equilibrium pressures during dehydrogenation (a high pressure for MgH_2_ and low pressure for Mg_2_FeH_6_). However, only one equilibrium pressure was observed for hydrogenation from a 2Mg-Fe stoichiometric mixture.

Puszkiel et al. [224] proposed a mechanism of Mg_2_FeH_6_ formation under equilibrium conditions for a 2MgH_2_-Fe powder mixture. During BM of the mixture, Mg_2_FeH_6_ and MgH_2_ were found to exist together from the beginning, but Mg_2_FeH_6_ was a result of reactions between elemental Fe and Mg. The formation of MgH_2_ was enhanced by the presence of Fe, but MgH_2_ did not take part as an intermediate in the formation of Mg_2_FeH_6_. It was noted that the partial formation of Mg_2_FeH_6_ could be attributed to the presence of MgH_2_ around Fe-rich particles, which acted as a solid-solid diffusion barrier. Polanski et al. [207] synthesized Mg_2_FeH_6_ by a two-step method with the use of a magnesium hydride powder and an iron powder as the starting materials. The synthesis first comprised mechanical milling of the MgH_2_ and Fe mixture over a short period in argon, followed by sintering at a high hydrogen pressure (>8.5 MPa). The samples were milled for different times (up to 3 h), but the best results were obtained for the samples milled for 2 h after annealing at 500 °C. Furthermore, the Mg_2_FeH_6_ synthesis/decomposition mechanisms were investigated. This time, the direct reaction of the MgH_2_ phase with Fe was shown to involve a two-step reaction: synthesis and then hydrogenation. The precursor for the formation of a ternary hydride (from the MgH_2_-Fe mixture) was MgH_2_. On the other hand, the decomposition path did not involve the above two-step reaction.

The two abovementioned cases prove that the formation of Mg_2_FeH_6_ may occur both as a reaction of magnesium hydride with iron and as a reaction of magnesium and iron in the presence of hydrogen, depending on the conditions. Decomposition, however, proceeds in only one way, whereby the ternary hydride is decomposed directly to two metals, and no magnesium hydride formation has ever been observed. Moreover, direct decomposition is quite understandable when considering the thermodynamics of both hydrides. Because the ternary hydride is more stable than magnesium hydride, under the pressure and temperature conditions that allow the decomposition of Mg_2_FeH_6_, no existence of magnesium hydride is possible.

#### 3.2.3. Light Complex Hydride Additives

**Figure 15:**
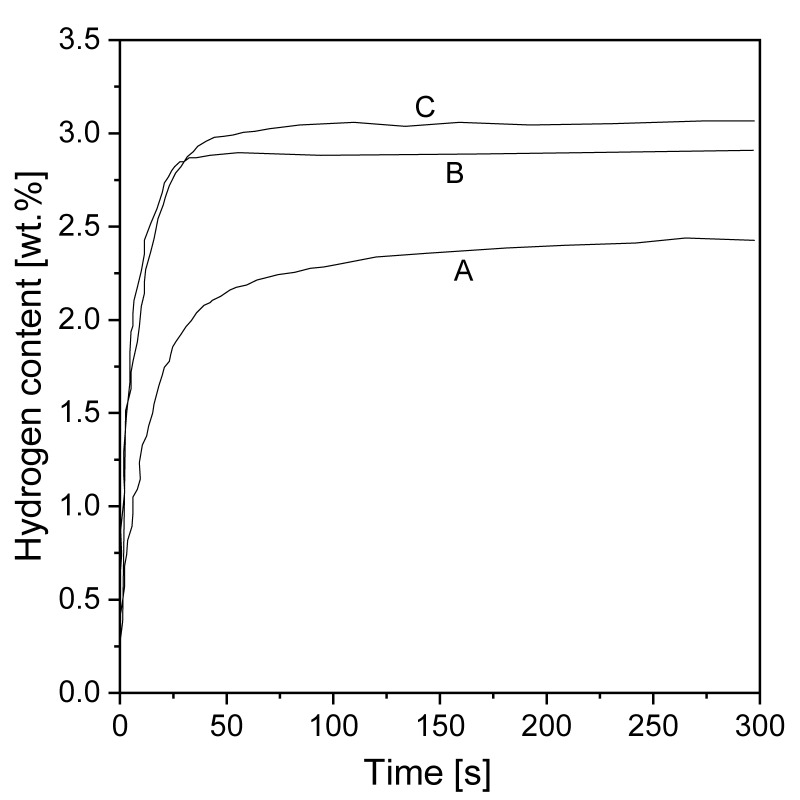
Absorption kinetics at 623 K for the first hydrogenation at 3 MPa. (A) 2MgH_2_ + Fe ball milled for 10 h, (B) 2MgH_2_ + Fe ball milled for 10 h and further milled with LiBH_4_ for an additional 1 h, and (C) 2MgH_2_ + Fe + LiBH_4_ ball milled for 10 h. The graph is based on [231].

Several reports have shown that the decomposition temperature and sorption of ternary hydride kinetics may be improved. The beneficial effects of additives on Mg_2_FeH_6_ have been broadly investigated. Puszkiel and Gennari [227] showed that a composite mixture of Mg-Fe powders doped with 10 mol% LiBH_4_ did not change the thermodynamics but led to a much higher capacity and faster kinetics than those of an undoped composite. Much research has focused on the interaction between MgH_2_ and light complex hydrides (such as LiBH_4_ and LiNH_2_). It was found that the sorption properties improved with the use of LiBH_4_ [228,229,230], whereas other Mg-based hydrides were treated rather as catalytic additives. Gosselin et al. [231] suggested that LiBH_4,_ instead of acting as a catalyst for the synthesis of Mg_2_FeH_6_, inhibited its formation and promoted the existence of MgH_2_ during BM in an argon atmosphere. This effect resulted in obtaining the fastest kinetics for the MgH_2_-Fe sample without the borohydride dopant. However, the absorption situation was slightly different. Figure 15 shows the absorption kinetics curves for the three synthesized materials following the desorption of the ball-milled samples. Clearly, the samples with LiBH_4_ possessed not only faster kinetics but also a higher capacity. This effect was, however, hard to explain since the materials without the additive in such stoichiometry should have more than 5% capacity. Thus, the reason for this behavior has not been fully explained.

#### 3.2.4. Mg_2_FeH_6_ as a Catalyst

In some cases, the Mg_2_FeH_6_ phase is used not as a base compound but as a catalyst [232,233]. In an initial study, the hydrogen storage properties of a 5LiBH_4_ + Mg_2_FeH_6_ system were investigated. Magnesium-iron hydride was synthesized using 2Mg-Fe powders as starting materials. During low-pressure decomposition, improved dehydrogenation was noted as a result of the in situ formation of Mg and Fe particles. These particles were products of the self-decomposition of magnesium-iron hydride before the dehydrogenation of LiBH_4_. A three-step desorption was observed with a total hydrogen desorption capacity of 8 wt.% over 300 min. Even after several cycling tests, the dehydrogenation capacity for the mixture was approximately 6.5 wt.% at 450 °C. Moreover, the MgH_2_-to-Mg_2_FeH_6_ transformation did not fully occur, which caused a decrease in the hydrogen sorption capacity and in the kinetics of the final dehydrogenation step as a result of the formation of Fe-boride, which acted as a formation nucleus for MgB_2_.

Later, the dehydriding properties of LiBH_4_ with Mg_2_FeH_6_ were examined by Li et al. [233]. Mg_2_FeH_6_ was synthesized by the mechanical milling of magnesium hydride and elemental Fe powder. Subsequent heat treatment was performed in a hydrogen atmosphere, and then the materials were mixed with LiBH_4_. The addition of magnesium-iron hydride to LiBH_4_ caused simultaneous dehydriding reactions, although their dehydriding temperatures were distinctly different by approximately 73 °C. The peaks occurred at 225 and 455 °C for pure Mg_2_FeH_6_ and LiBH_4_, respectively. Mixing these two compounds resulted in hydrogen desorption from 255–305 °C. With a low magnesium-iron hydride concentration (according to the chemical formula of the compound xLiBH_4_ + (1 − x)Mg_2_FeH_6_), the dehydriding temperature increased, and the one-step reaction transformed into a multistep reaction.

### 3.3. Reactive Ball Milling

**Figure 16:**
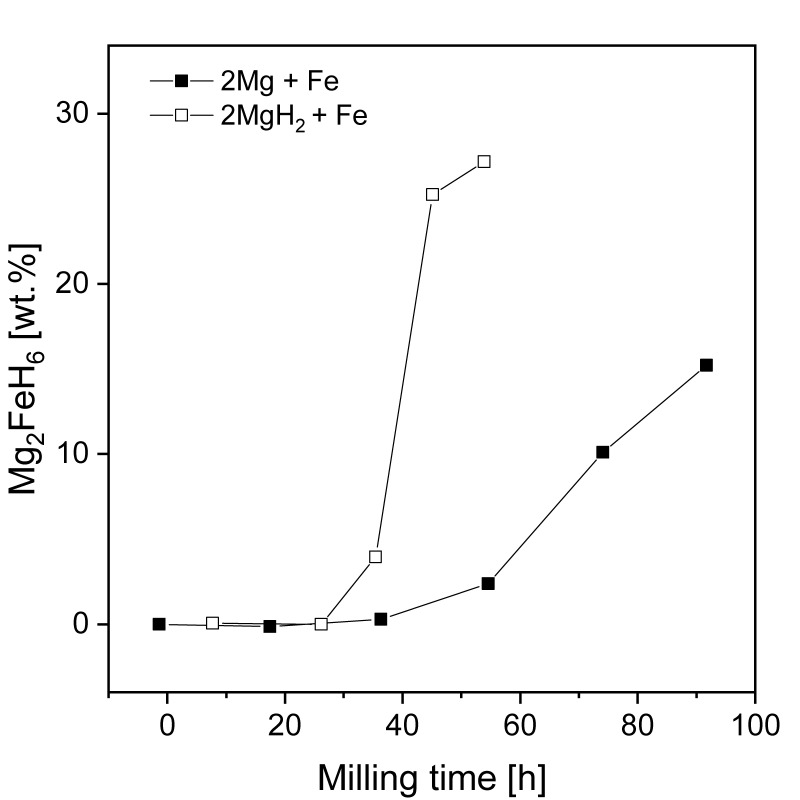
Amount of Mg_2_FeH_6_ as a function of milling time for two mixtures: 2Mg + Fe and 2MgH_2_ + Fe. The curves are based on [39].

MgH_2_ was milled together with Fe in a H_2_ atmosphere at room temperature for the first time by Huot et al. [222]. Notably, it took 10 h to observe the presence of Mg_2_FeH_6_. The MgH_2_ phase disappeared after 30 h of BM, replaced by Mg_2_FeH_6_ and pure magnesium. An increase in milling time (up to 60 h) did not change the distribution of existing phases. The hydride efficiency after 60 h of milling was 56%. Furthermore, compared with sintered samples, the as-prepared samples demonstrated better reversibility and decreased hydride capacity after cycling (reduction by ~5%). Later, Castro et al. [39] performed 140 h of total milling of a 2MgH_2_ + Fe mixture, obtaining a Mg_2_FeH_6_ yield no higher than 15.6 wt.% at 100 h. Further increases in milling time caused the Mg_2_FeH_6_ peak intensity to decrease and the MgO peaks to increase. After 10 h of milling, one endothermic reaction was noted at 370 °C. Samples milled for 60 h were characterized by a two-step reaction, but increased milling time (80–100 h) again showed a one-step reaction. Due to a decrease in particle size and more uniform mixing between MgH_2_ and Fe particles, the endothermic peaks shifted toward lower temperatures. In the same research, a 2Mg + Fe mixture milled under similar conditions demonstrated an almost two-fold higher reaction yield (~30 wt.%). Both values were determined from the area under the DSC curves (Figure 16). The decomposition temperature was found to be ~50 °C lower than that for the 2MgH_2_ + Fe mixture for short milling times. For long milling times, the peak angle positions did not change significantly, but the gap between the two mixtures was ~25 °C. Similar research was reported by Asselli et al., in which milling and reactive mechanical milling (RMM) were used to synthesize 2Mg + Fe and 2MgH_2_ + Fe mixtures [234]. The hydrogen atmosphere caused the particle size to decrease, which improved homogeneity and resulted in a phase abundance of up to ~88 wt.% Mg_2_FeH_6_ (for a 2MgH_2_ + Fe mixture synthesized at a certain hydrogen pressure).

Herrich et al. [40] used an RBM method at room temperature with mixtures of MgH_2_ and Fe powders at different atomic stoichiometries. The effect of different addition amounts of Fe on the synthesized powders was studied. The hydrogen storage capacity of the material after 80 h milling was 5.2 wt.%., and ~78 wt.% Mg_2_FeH_6_ was achieved, as well as ~20 wt.% pure Fe, 0.5 wt.% magnesium hydride and ~1.5 wt.% MgO. Magnesium oxide was most likely present due to the presence of oxygen during milling. Finally, a 92 wt.% Mg_2_FeH_6_ yield was achieved by changing the Fe concentration during milling. The decomposition temperature was established as 355 °C, which is significantly lower than the previously reported value of 428 °C [222]. During reabsorption at 1 MPa of hydrogen pressure and 300 °C, the hydrogen uptake decreased to ~3 wt.%. This decrease was a result of the disproportionation of Mg_2_FeH_6_ into elemental magnesium and iron upon desorption and the formation of magnesium hydride during rehydrogenation. The authors claimed that the synthesis rate of magnesium-iron hydride was not enhanced by the temperature of the vial but rather the high energy input during milling. With the above method, a reaction yield of up to 92 wt.% was achieved. Another study [235] consisted of premilling Mg and Fe powder in an inert atmosphere (argon) and further BM at a certain hydrogen pressure. Unfortunately, the efficiency was relatively low (with the highest yield being ~34 wt.%) due to the presence of unreacted Fe. The premilling of elemental powders can accelerate Mg_2_FeH_6_ formation, but such premilling needs to be optimized because a 90 wt.% Mg_2_FeH_6_ phase abundance was achieved in a later work [205,236].

An investigation of the microstructure and the hydrogenation behavior of magnesium-iron hydride was conducted with mechanically alloyed 2Mg-Fe [217], and a broad description of the Mg_2_FeH_6_ phase forming process was provided. All powders were synthesized in a 0.88 MPa hydrogen atmosphere with different alloying times (in the range of 18–270 h). The presence of different phases was noticed after continuous and sequential milling. For all powders, only a single endothermic peak was visible, even for the material containing both β-MgH_2_ and Mg_2_FeH_6_ phases (in the temperature range of 274–289 °C). The TGA curves showed a maximum weight loss of 3.7 wt.% for the 2Mg-Fe mixture ball milled for 270 h.

#### 3.3.1. Improvements in Reaction Yield

In the context of Mg_2_FeH_6_ synthesis, most research is focused on improving the reaction yield. Since a yield of 55 wt.% had been previously achieved by a nonreactive BM method, Gennari et al. [38] attempted Mg_2_FeH_6_ synthesis from a 2Mg + Fe mixture by RBM without a subsequent sintering step. The first Mg_2_FeH_6_ peak was visible in the XRD pattern after 40 h of milling. After 60 h of milling, no MgH_2_ peak was visible, but the presence of metallic Fe with Mg_2_FeH_6_ was noted. According to the authors, a decrease in crystallite size, amorphization, and/or plastic deformation occurred by the end of the process, as indicated by peak broadening. DSC analysis showed an endothermic peak at 321 and 303 °C after 10 and 30 h of milling, respectively, proving the decomposition of MgH_2_. The decomposition temperatures shifted to low values of approximately 100 °C (in comparison to that of pure magnesium hydride synthesized by MA) due to the presence of Fe, particle size reduction, increased surface area, and morphological changes. The authors observed that the presence of Fe had an influence on the thermal stability of magnesium hydride and acted as a catalyst in the desorption process for samples with short milling times. Hence, this approach caused a decrease in activation energy of approximately 60 kJ/mol. After 60 h of milling, the Mg_2_FeH_6_ yield was approximately 30 wt.%, and an increase in milling time did not further increase the yield. It was shown that the two-step formation of Mg_2_FeH_6_ involved the presence of the MgH_2_ phase. The experiment proved that MgH_2_ was unstable upon the formation of Mg_2_FeH_6_. During the first 40 h of milling, the amount of MgH_2_ increased to 23 wt.% and then dropped significantly. From that moment, Mg_2_FeH_6_ started to appear.

The synthesis of Mg_2_FeH_6_ from elemental powders was performed by Gennari et al. [38] with a single-step reaction that required no further sintering. The investigation showed that the MgH_2_ phase peak increased up to 60 h of milling, at which point Mg_2_FeH_6_ started to form. A further study [39] found that the synthesis of Mg_2_FeH_6_ proceeded by the formation of MgH_2_ followed by the reaction of this hydride with Fe. XRD peaks indicated that with continuous milling, the MgH_2_ phase disappeared due to Mg_2_FeH_6_ formation. The maximum yield of ball-milled Mg_2_FeH_6_ in an inert atmosphere was estimated to be 15.6 wt.% after 100 h, which was significantly lower than the value observed for a mixture synthesized in a hydrogen atmosphere. Moreover, the whole process lasted longer in an inert atmosphere. Mg_2_FeH_6_ synthesis from 2MgH_2_ + Fe resulted in a shorter synthesis time and a higher reaction yield as an effect of prolonged surface contact between Mg and Fe. Huen et al. [236] performed Mg_2_FeH_6_ synthesis by milling pure Mg with Fe powder. The product was intended for use as a conversion-type anode for solid-state lithium batteries with a LiBH_4_ electrolyte. The as-synthesized sample composition was 92 wt.% Mg_2_FeH_6_ and 8 wt.% unreacted Fe.

#### 3.3.2. Nanostructurization Effect

As in the binary magnesium hydride case, the main focus of nanostructurization is to obtain improved solid-state hydrogen storage materials. Many attempts have been made to decrease crystallite size to achieve great improvements in the hydrogen absorption/desorption rates of magnesium-iron hydrides synthesized by RBM methods [205,208,215,235,237,238].

A natural continuation of research by Li and Varin [235] was conducted to study the synthesis of nanomagnesium-iron hydride by RBM in high-energy impact mode [237]. Two types of RMA were applied to Mg and Fe powders: sequential and continuous. Using the continuous RMA mode was predicted to inhibit MgO formation and provide a much higher yield because it had been proven that MgO is a primary factor that suppresses magnesium-iron hydride formation. After continuous milling for 270 h, ~57 wt.% magnesium-iron hydride was noted (in comparison to ~34 wt.% obtained in previous work [235]). The desorption (3.13 wt.% H_2_ in the DSC test and 2.83 wt.% in the TGA test) temperatures were in a relatively narrow range of 200–300 °C, which corresponded to 10–20 min of reaction. The size of the nanograins was estimated to be in the range of ~5–13 nm. Samples milled in sequential mode desorbed just under half the hydrogen content desorbed by the samples synthesized in continuous mode, but no hydrogen desorption was noted in the 250–90 °C temperature range in sequential mode.

Yan Wang et al. [205] prepared nanocrystalline Mg_2_FeH_6_ by BM a 3Mg + Fe mixture at room temperature and 0.55 MPa hydrogen pressure. Structural analysis was conducted and showed a chrysanthemum-like nanostructure composed of aggregated spherical Mg_2_FeH_6_. The total mass loss was 5.15 wt.%, which was correlated to hydrogen desorption from the magnesium-iron hydride phase in a temperature range of 60 to 340 °C. The same experiment was carried out by Asseli et al. [238] but at 3 MPa hydrogen pressure and with the additional synthesis of a 2Mg-Fe mixture. Under the same conditions, the synthesized 3Mg-Fe and 2Mg-Fe mixtures had hydrogen gravimetric density values of ~5.2 wt.% and ~3.5 wt.%, respectively. The higher hydrogen capacity of the 3Mg-Fe mixture was a result of the presence of α-MgH_2_ and complete reaction between the metallic elements and hydrogen. In the case of the 2Mg-Fe mixture, a 65 wt.% Mg_2_FeH_6_ abundance was noted.

A highly nanocrystalline mixture of MgH_2_ and Mg_2_FeH_6_ was synthesized by RBM of 3MgH_2_-Fe (sample A) and 40MgH_2_-Fe (sample B) at room temperature and 3 MPa hydrogen pressure [215]. The crystallite dimensions were estimated as ~10 nm and increased with increasing cycling temperature. The same dependence was noticed for the sorption kinetics. The differential thermal analysis (DTA) results (performed before any hydrogenation cycling) proved that a two-step reaction occurred for sample A (~4.1% weight loss in the 210 to 300 °C temperature range) and that a one-step reaction occurred for sample B. The two-step desorption was a consequence of the subsequent dehydrogenation of magnesium hydride and Mg_2_FeH_6_ in materials obtained either during the BM of MgH_2_ and Fe or by the direct hydrogenation of these powders. The total amount of hydrogen stored in the sample was ~4.8 wt.%. The above results indicated that the composition of sample A was ~30 wt.% magnesium hydride and ~44 wt.% magnesium-iron hydride at 335 °C (first peak) and then changed to ~24 wt.% magnesium hydride and ~54 wt.% magnesium-iron hydride at 390 °C (second peak).

A breakthrough was made by Brutti et al. [239]. A mixture of 2MgH_2_ + Fe was synthesized by two methods: RBM at a high hydrogen pressure (5 MPa) and hand grinding. To obtain full conversion, various hydrogenation temperatures were implemented. The full magnesium-iron hydride conversion, calculated from the widths of the plateaus at various temperatures, was higher than 97% at 485 °C. Decreasing the iron particle size caused the Mg and Fe powders to mix more easily. Much shorter diffusion occurred, which was beneficial for producing a high magnesium hydride abundance. Furthermore, increased cycling temperature promoted Mg_2_FeH_6_ formation (the magnesium hydride concentration at 485 °C was lower than 3%). A total hydrogen capacity of up to 5.4 wt.% at 485 °C was noted for the ball-milled sample, which corresponded to pure Mg_2_FeH_6_.

#### 3.3.3. Cycling Stability

In general, Mg-Fe mixtures seem to be extremely stable upon cycling. However, in some cases, capacity loss has been observed. The evolution of the hydrogen storage capacity throughout hydrogenation-dehydrogenation cycles was measured by Puszkiel et al. [218] with three different Mg-Fe ratios. The measured hydrogen capacity for a 15Mg-Fe mixture before and after cycling at 375 °C was 6.6 and 4.3 wt.% H_2_, respectively. Two effects could be responsible for the decreased hydrogen capacity upon cycling: Mg evaporation under moderate pressure conditions and Mg_2_FeH_6_ formation. The cycling process did not affect the hydrogenation kinetics behavior, but the dehydrogenation kinetics properties worsened. Unfortunately, the relative amount of the Mg_2_FeH_6_ phase before and after cycling (at 375 °C) was 23 and 27 wt.% for 2Mg-Fe, respectively, and 0 and 11 wt.% for 15 Mg-Fe. The other remaining phases were magnesium hydride and some unreacted pure Fe. On the other hand, no formation of Mg_2_FeH_6_ was observed by Varin et al. [240] from BM 2Mg-Fe mixtures (in only a hydrogen atmosphere or with additional prealloying in an argon atmosphere). The samples were “aged”, which meant they were stored for approximately four months in an argon atmosphere and examined after that time. The authors suggested that initially, crystalline Mg became amorphous with increasing milling time (β-MgH_2_ amorphization). Weight losses on the order of ∼2–4 wt.% and a single DSC peak corresponding to the release of hydrogen, mostly from the amorphous hydrides, were observed. Exposing the ball-milled mixture to moisture showed for the first time that β-MgH_2_ underwent hydrolysis into Mg(OH)_2_, which was related to the high weight loss (up to 10 wt.%). Urbanczyk et al. performed twenty-three dehydrogenation/hydrogenation cycles in a tube storage tank with a 2Mg-Fe powder mixture [219]. The material was tested at temperatures between 395 and 515 °C and pressures between 1.5 and 8.6 MPa. Phase analysis showed mainly Mg_2_FeH_6_ with a small amount of MgH_2_ and pure Fe, but the hydrogen capacity was only 3.8 wt.%.

## 4. Mg_2_NiH_4_

Another popular magnesium-based hydride is Mg_2_NiH_4,_ which was first reported by Reilly and Wiswall [31]. The precursor Mg_2_Ni phase existed in the Mg-Ni binary system, and the hydride had a theoretical hydrogen gravimetric density of 3.6 wt.% with at least two distinct polymorphs [241]. The dissociation enthalpy was −64 kJ/mol H_2_ [38,241], which made Mg_2_NiH_4_ less stable than magnesium hydride (a higher equilibrium pressure at the same temperature). The first reaction path provided by Reilly and Wiswall [31] turned out to be reversible:

Mg_2_Ni + 2H_2_ ⇄ Mg_2_NiH_4_ (2)

It has been reported that Mg_2_Ni can be produced by metallurgical methods, both pure and with intentional excess of magnesium [242]. Even if produced as a homogenous, almost single-phase material, it still needs activation at elevated temperature (~300 °C) before it can absorb hydrogen from the gas phase [243,244]. Usually, this procedure first requires 10–20 absorption/desorption cycles. Therefore, in 1995, Singh and Zaluski [245,246] used MA for the first time to produce Mg_2_Ni. Mg_2_NiH_4_ had a lower hydrogen desorption temperature (250 °C) than magnesium hydride and faster kinetics [241]. Further examination of this material proved that Mg_2_NiH_4_ exhibited changes in color with even a small microstructure change, thus exhibiting surface sensitivity. These differences were found to influence the desorption kinetics of the hydride, which was examined and described by Rönnebro [247]. Javadian et al. [248] successfully nanoconfined (for the first time) a LiBH_4_-Mg_2_NiH_4_ reactive hydride composite into two types of mesoporous carbons. The results were compared to those of a previous synthesis by ball milling performed by Vajo et al. [249]. According to the results, faster kinetics of hydrogen desorption were noted with a progressive loss of capacity upon cycling.

Mg_2_NiH_4_ structures have been identified as ordered low-temperature and disordered high-temperature phases with C2/m and C2/c space groups, respectively [241]. The high-temperature phase has a cubic structure (pseudo-CaF_2_ type). The Ni atoms are arranged on the cube edges and the cube face centers. The magnesium atoms have tetragonal positions [250], and the low-temperature phase has a monoclinic structure [250]. Despite many years of investigations, this complex hydride is still not fully known, but some research proved that several polymorphic phases exist (two low-temperature and two high-temperature phases). Increasing the temperature above 235 °C results in a phase transition [241,251,252]. In the low-temperature phase, the hydrogen atoms in the NiH_4_^4−^ complexes are rigid and have an orderly arrangement. In the high-temperature phase, they start to demonstrate reorientation motion around the central Ni atom. Cooling the hydride to room temperature leads to the introduction of a microtwinned form [43,253,254,255].

The transition from the low-temperature phase to the high-temperature phase was first noticed in 1979 [250]. Further analysis showed that the phase transition from the high-temperature to the low temperature phase occurred at ~235 °C [256]. Polanski et al. [254] synthesized cubic Mg_2_NiH_4_ with a yield of ~90% from a mechanically milled MgH_2_ and Ni mixture at a molar ratio of 2:1 in an argon atmosphere with subsequent sintering and observed the product in situ by synchrotron powder X-ray diffraction (SRPXD). The hydride had a polymorphic form after cooling to room temperature. Subsequent heating at high hydrogen pressure (10–12 MPa) initiated the formation of the first high-temperature structure, which was followed by hydride decomposition with Mg_2_Ni formation. The synthesized ternary hydride had nanoscale grains and decomposed in a one-step reaction upon heating in a helium atmosphere.

Mg_2_NiH_4_ has a hydrogen volumetric capacity 40% lower than that of Mg_2_FeH_6_ and a lower theoretical gravimetric density (3.6 wt.% in comparison with 5.6 wt.%). Currently, Mg_2_NiH_4_, similar to MgH_2_ and Mg_2_FeH_6,_ is not the first material considered for potential use in mobile applications because of its relatively low hydrogen capacity and high desorption temperature.

### 4.1. Mg_2_NiH_4_ Synthesis/Mechanical Modifications

Among magnesium-based alloys, Mg_2_Ni is another attractive option due to its relatively high capacity and favorable thermodynamics. However, the technological application of materials with a Ni component is limited by their cost. Much research has been devoted to using a combustion synthesis method for Mg_2_NiH_4_ production [257,258,259,260], and combustion synthesis remains the most popular way of producing this particular complex hydride. On the other hand, together with other complex hydrides (Mg_2_FeH_6_ and Mg_2_CoH_5_), Mg_2_NiH_4_ can be synthesized by using pure metals with hydrogen gas by BM or by sintering in a hydrogen atmosphere at high hydrogen pressure/high hydrostatic pressure. Mg_2_Ni absorbs hydrogen at moderate temperatures and pressures, and the hydride Mg_2_NiH_4_, with a 3.6 wt.% hydrogen capacity, can be formed [245]. However, this compound still desorbs hydrogen at relatively high temperatures (above 220 °C), which is problematic. Hydrogenation usually occurs at 250–350 °C and 1.5–5 MPa hydrogen pressure [246].

The crystalline Mg_2_Ni alloy produced by BM is characterized by better surface properties than those of corresponding materials prepared by conventional metallurgical methods [246]. The hydrogenation enthalpy of Mg_2_NiH_4_ (−64.5 kJ/mol H_2_) is lower than the enthalpy of MgH_2_ formation (−75 kJ/mol H_2_) [42,261]. Under normal conditions, Mg_2_NiH_4_ is too stable and useless for room temperature applications. The main goal is to decrease the stability of Mg_2_NiH_4_ to produce a suitable material for practical hydrogen storage.

#### 4.1.1. Synthesis Mechanism

Baum et al. [262] performed RBM in a hydrogen atmosphere of a pure elemental 2Mg-Ni powder mixture. The total amount of hydrogen absorbed did not correspond to ideal stoichiometry. During the first 2 h of the experiment, stable but slow hydrogen absorption was observed. The XRD patterns showed both metallic Mg and β-MgH_2_ phases. The reaction between Mg_2_Ni and H_2_ resulted in the existence of both phases: Mg_2_NiH_4_ and Mg_2_Ni. Mg_2_NiH_4_ reversibly formed from Mg_2_Ni, which made it the only transition metal complex hydride with a corresponding hydrogen-free intermetallic precursor. The decomposition of Mg_2_NiH_4_ started at 274 °C, which was visible as an exothermic peak.

The reactive synthesis of Mg_2_NiH_4_ has been previously achieved. However, with the use of pure H_2_ gas remains problematic. Mg_2_FeH_6_, Mg_2_CoH_5,_ and Mg_2_NiH_4_ synthesis was performed by grinding metal powders at 9 MPa H_2_ pressure [220], which resulted in nanocrystallites (~10 nm). Hydriding combustion synthesis was also attempted. This method has been combined with mechanical milling for the production of magnesium-based hydrogen storage alloys [263]. BM in an inert atmosphere followed by hydrogenation has received great attention as a pioneering technique for the preparation of nanocrystalline hydrogen storage materials such as Mg_2_NiH_4_. Ayoagi et al. [264] attempted hydrogenation on previously ball-milled Mg_2_Ni. The unmilled sample did not absorb hydrogen at all, while even a short milling time led to hydrogen absorption. New surfaces helped to improve the hydrogen absorption rate, which occurred even at room temperature. With an increase in milling time, the particle size decreased and reached ~5 µm. Melting and crushing the Mg_2_Ni powder, followed by hydrogenation, led to Mg_2_NiH_4_ formation [204]. Very fast hydrogenation at relatively low temperatures (230–330 °C) was correlated with hydrogen capacity losses (~3 wt.% at 0.04–0.28 MPa).

Sashi et al. [180] performed studies on the catalytic effect of transition metals (Ti, Fe, and Ni) on the hydrogen storage properties of nano-MgH_2_. Mechanical milling of MgH_2_ separately from Ni showed XRD peaks corresponding to the Mg_2_NiH_4_ phase. On the other hand, milling magnesium hydride together with all the above elements did not cause the Mg_2_NiH_4_ (or Mg_2_FeH_6_) phase to form due to the presence of Ti. According to the authors’ statements, Ti had little to no solubility with Mg. Hence, it partially screened the diffusion of Ni (or Fe) on the surface of magnesium hydride to form ternary hydrides.

**Figure 17:**
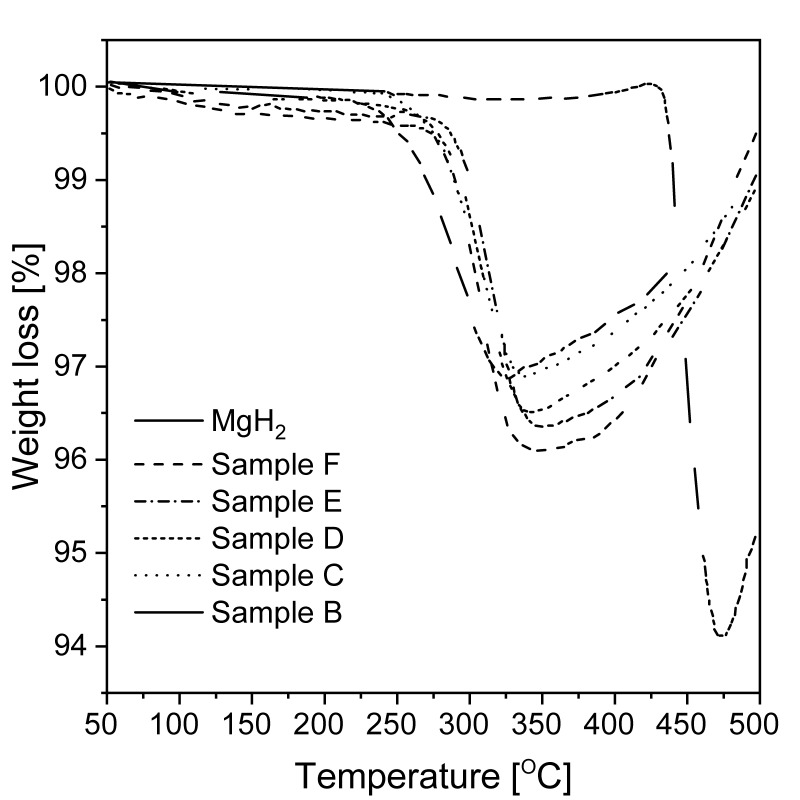
TGA curves for the synthesized samples and MgH_2_. The graph is based on [265]. B–F represent different technological parameters and compositions (for details, please see [265]).

Martinez-Coronado et al. [265] showed a simplified method of milling magnesium hydride with pure metallic nickel in a nitrogen atmosphere at room temperature. Different MgH_2_ to Ni ratios (2:1, 2:0.9 and 2:0.8) as well as different milling times (4–16 h) were examined (samples B-F). A yield of up to 93.2% Mg_2_NiH_4_ was achieved with hydrogen capacity values between 2.8 and 3.24 wt.%, which were lower than the theoretical value of 3.6 wt.% (Figure 17). It was noted that a H_2_ atmosphere was not needed for hydride synthesis. The desorption process started at a temperature between 217 and 260 °C. The authors state that the complex hydride decomposed into elemental magnesium and nickel and underwent further oxidation, regardless of the presence of a reducing H_2_/N_2_ flow. However, this statement seems to be very controversial given that this hydride usually decomposes into a Mg_2_Ni intermetallic phase [254]. An endothermic peak associated with the decomposition of Mg_2_NiH_4_ was observed at temperatures between 267 and 297 °C. It is worth noting that the character of the TGA curves (a steep increase in mass) (Figure 17) showed that it was very likely that an oxidation reaction with nitrogen took place during the decomposition process. In this case, the validity of the obtained results is debatable and may not be trustworthy.

RMM has also been used to synthesize a Mg_2_Ni-H system [266]. Mg_2_Ni was milled in a hydrogen atmosphere. The achieved hydrogen capacity was 1.6 wt.% H_2_ without changing the crystal structure (Mg_2_Ni type). The hydriding properties were described as most likely reversible at temperatures below 200 °C, whereas above 200 °C, the disordered intergrain area changed into a crystalline phase.

#### 4.1.2. Microtwinning Phenomena

**Figure 18:**
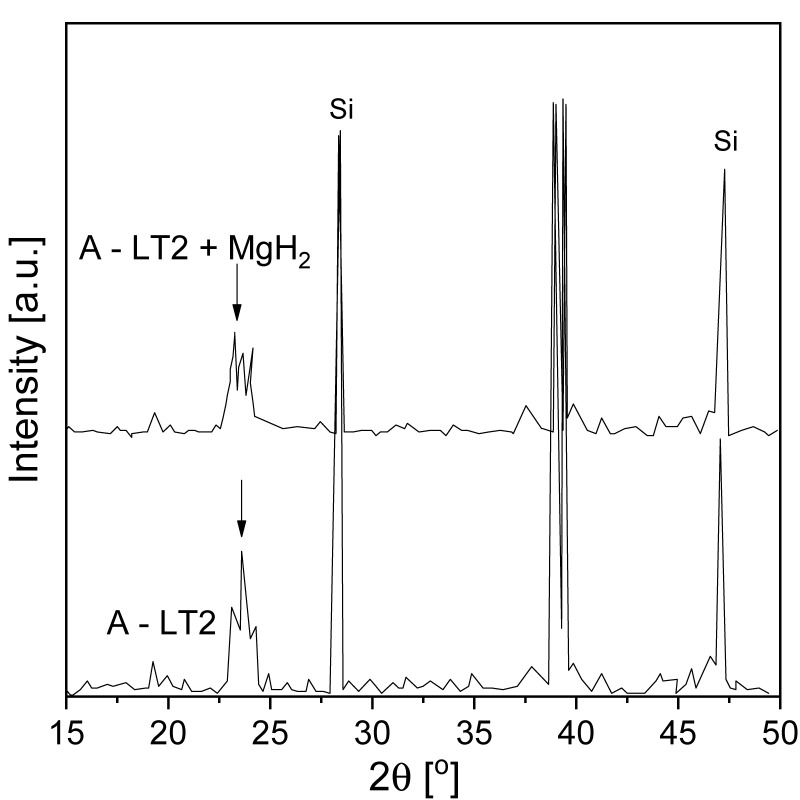
XRD profiles of ball-milled Mg_2_NiH_4_ with the low-temperature phase visible. The graph is based on [267].

Blomqvist [43] carried out the synthesis of Mg_2_NiH_4_ by using Mg_2_Ni alloys manufactured by different methods. Cooling the high-temperature phase of Mg_2_NiH_4_ to room temperature was followed by the formation of a low-temperature phase that exhibited microtwinning. Rehydrogenation led to a decrease in the amount of the microtwinned phase. This result was consistent with the phase change, which was revealed by the color change of the powder. No impurity phase of MgH_2_ was observed. The phase transition kinetics were strongly dependent on the microtwinned sample content. It was proven that with respect to the microtwinned phase, not only was the thermal history of the sample important but also the presence of free magnesium (an unwanted dopant changing the color of the low-temperature phase) in the compound. A lower synthesis temperature was suggested to avoid the stabilizing effects of Mg_2_NiH_4_. Both the microtwinned phases and the Mg dopants acted the same way—they behaved as nucleation centers, which improved the transition between the low- and high-temperature phases [267]. These stabilization mechanisms competed with each other, but the details about this behavior remain unclear. When the low-temperature Mg_2_NiH_4_ phase was synthesized by MA with additional magnesium hydride, the amount of microtwinning after rehydriding was reduced, and the color of the powder was orange. XRD profiles are presented in Figure 18. The MgH_2_ dopant in the mixture also influenced the appearance of the powder, changing the color from brownish-gray to orange.

#### 4.1.3. Carbon Additives

**Figure 19:**
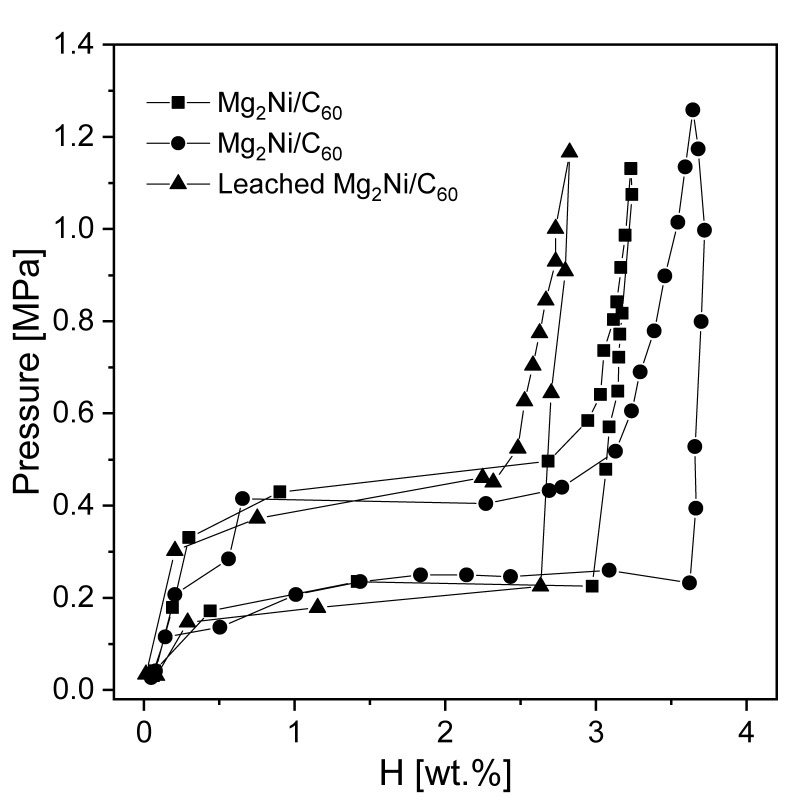
Pressure-composition isotherms at 300 °C of Mg_2_Ni and Mg2Ni/C_60_ compounds before and after leaching. The graph is based on [269].

Nohara et al. [268] proved that the surface modification of BM Mg_2_Ni with a graphite additive improves the hydrogen capacity and hydrogenation/dehydrogenation cycling as a result of the increased number of active sites for absorption. Another explanation involved the formation of chemical bonds between magnesium and graphite. The rate of hydrogen absorption turned out to be barely dependent on the particle size in that case, which indicated the importance of surface modification in the context of hydrogen absorption. Another attempt to examine the hydriding behavior of various Mg_2_Ni alloys was provided by Bouaricha et al. [269]. Mg_2_Ni was ball milled with 5 wt.% fullerene (C_60_) and leached in an organic solvent, which caused crystallite size refinement (in a range of 11–19 nm). Figure 19 presents pressure-composition isotherms of Mg_2_Ni and Mg_2_Ni/C_60_ samples (before and after leaching). All curves show a plateau pressure at ~2 or 4 atm (0.2 or 0.4 MPa) during desorption and absorption, respectively. On this basis, it can be concluded that neither additional milling with fullerene nor leaching treatment in toluene caused any modifications leading to improved thermodynamics properties. Even a small amount (5 wt.%) of C-containing compounds (such as fullerene, graphite, and Vulcan) causes an ~5% decrease in (H/M)_max_. On the other hand, the time needed to reach complete desorption was reduced by 2–3 times. Properly chosen C compounds can be used to increase the specific surface area but have no effect on hydrogen desorption kinetics. On the other hand, changing the surface area can be helpful in the preparation of hydrogen absorption materials that are more stable during cycling. Similar research results were reported by Bobet et al. [190]. The authors suggested that during the process of hydrogenation of Mg_2_Ni with a modified graphite compound, two different phenomena occur. One phenomenon is related to increased gas-solid surface contact caused by the destruction of the oxide layer and its partial blockage by graphite.

## 5. Mg_2_CoH_5_

Mg_2_CoH_5_, a complex hydride, is analogous to the previously mentioned hydrides. However, it is an attractive hydride for several applications and is an interesting compound on its own. Its gravimetric hydrogen capacity is 4.5 wt.%, and theoretically, it can be used as a stationary hydrogen storage and thermal energy storage medium [270]. The main advantages of this hydride are its relatively high gravimetric hydrogen capacity and good kinetics for hydrogen sorption. However, regardless of the cost of the cobalt itself, as in the case of most ternary hydrides, the synthesis of Mg_2_CoH_5_ is a very interesting and not trivial process. There is no Mg_2_Co intermetallic compound in the equilibrium phase diagram of Mg-Co, but Mg_2_Co, MgCo, and MgCo_2_ have been proven to exist after the hydride decomposition of ternary hydrides [270]. The Mg-Co-H system is interesting mostly because of the different hydride phases existing as a function of pressure and temperature (Mg_2_CoH_5_, Mg_6_Co_2_H_11_, and MgH_2_) [271,272]. These hydrides are receiving attention due to their potential kinetic/thermodynamic properties associated with their synthesis techniques. The hydride Mg_2_CoH_5_ can be formed only by energetic techniques: sintering, mechanical milling, and reactive mechanical milling (RMM) under specific temperature and pressure conditions. The Mg-Co-H system allows the formation of two complex hydrides: tetragonal β-Mg_2_CoH_5_ and orthorhombic γ-Mg_6_Co_2_H_11_ [272,273]. However, other phases, namely MgH_2_ and Co with content percentages dependent on the experimental conditions, are also usually present [271,272]. The absence of stable precursors (Mg_2_Co or Mg_3_Co) and the simultaneous formation of MgH_2_ make the synthesis of a complex Mg-Co hydride (as a single-phase) from elemental Mg and Co theoretically difficult (similar to Mg-Fe).

Pioneering attempts to synthesize Mg_2_CoH_5_ and Mg_6_Co_2_H_11_ hydrides were performed by Zolliker et al. and Ivanov et al. [271,272] from 1985–1988, and the formation of Mg_2_CoH_5_ was reported for the first time. A cylindrical 2Mg–Co pellet mixture was sintered for several days between 415–445 °C and 4–6 MPa hydrogen pressure. To avoid the drastic experimental conditions used during sintering, an important improvement in the preparation of hydrides was achieved by RMM. With this technique, the raw materials were ground at a certain hydrogen pressure (up to 1 MPa) and room temperature to favor a gas-solid reaction during milling, thereby creating structural/surface defects and producing a refined material. The low-temperature hydride from the Mg-Co-H system was initially referred to as Mg_3_CoH_5_ and characterized by a hexagonal structure [272]. Cerny et al. [273] finally found that the structure was orthorhombic, with a stoichiometric composition of Mg_6_Co_2_H_11_.

From a technological point of view, the hydrides Mg_6_Co_2_H_11_ and Mg_2_CoH_5_ are attractive for storage applications due to their high gravimetric (4.0 wt.% and 4.5 wt.% hydrogen, respectively) and volumetric (>90 kg m^3^) hydrogen storage capacities. However, the complexity of the Mg-Co-H system, with different hydride phases and the absence of stable precursors, is the main reason for the lack of knowledge about this system. In addition, the absorption and desorption kinetics, as well as the hydrogen storage reversibility of Mg_6_Co_2_H_11_ and Mg_2_CoH_5,_ are still poorly understood.

### 5.1. Mg_2_CoH_5_ Synthesis/Mechanical Modifications

Since Mg and Co do not alloy at all in the H-free solid metallic state, one of the most common ways of preparing the complex metal hydride Mg_2_CoH_5_ is to sinter Mg and Co powders at high hydrogen pressures [274]. However, Mg_2_CoH_5_, similar to the hydrides mentioned above, can be synthesized by mechanical milling (RMM in a hydrogen atmosphere or simple mechanical milling in an inert atmosphere). Further sintering is also considered in some approaches (as performed by Huot et al. in [36] and [32,275]), but in most approaches, this step is neglected [33,34,276]. However, the above methods require considerable time (even several days), and the yield is relatively low (~50%).

Sintering was used for the first time by Ivanov et al. [272]. Pure magnesium and cobalt powders were ball milled for 2–3 days and then sintered. The hydriding process was retained regardless of the phase diagram. By measuring a series of pressure-composition isotherms, the enthalpy values of the two hydride phases (low- and high-pressure hydrides) were determined to be −70 and −79 kJ mol^−1^ H_2_, respectively, for hydrogen desorption. As reported by Zolliker et al. [271], Mg_2_CoH_5_ and its deuteride could be prepared by sintering previously ball-milled pure Mg and Co between 415 and 445 °C in a 4–6 MPa hydrogen (or deuterium) pressure atmosphere. The MgH_2_ and pure metallic Co phases, which remained unreacted, were removed. The enthalpy values were calculated to be 86 kJ/mol H_2_ (desorption) and -60 kJ/mol H_2_ (absorption). These data were in good agreement with the data provided by Ivanov et al. [272]. Furthermore, this result indicated the existence of hysteresis phenomena. The synthesized material was a black crystalline solid with a tetragonally distorted CaF_2_-type structure that transformed into a disordered cubic phase at 215 °C. However, when implementing the above approach during complex hydride synthesis, a reaction yield of lower than 30% was obtained.

A breakthrough in hydride technology was achieved by preparing hydrides via mechanical synthesis. To obtain Mg_2_CoH_5_, MA must be implemented: milling Mg and Co in an inert atmosphere followed by sintering [271], milling Mg and Co in a H_2_ atmosphere (RMA) [34], or milling MgH_2_ and Co in a H_2_ atmosphere [277]. The sintered material is exposed to drastic experimental conditions that are limited by the moderate hydrogen pressure and room temperature used in the RMA technique. Unfortunately, little research has been devoted to synthesis procedures involving BM designed to obtain Mg_2_CoH_5_ as the only ternary hydride.

A novel method of Mg_2_CoH_5_ synthesis was presented by Norek et al. [276]. A 2:1 MgH_2_-Co powder mixture was ball milled for 1 h in an argon atmosphere. Later, sintering at a high hydrogen pressure (>8.5 MPa) and heating from room temperature to 500 °C was applied. The relatively short milling time and high H_2_ pressure prevented MgH_2_ decomposition, which increased the reaction rate and final reaction yield. The authors suggested that the yield of the reaction (~90 wt.%) was connected to the amount of synthesized sample due to the heat generated by the reaction itself. The increased yield was also caused by high pressure, which helped to avoid magnesium hydride decomposition. No pure Mg was observed before the formation of Mg_2_CoH_5_ or any MgH_2_ phases, which reacted with cobalt during the reaction. The authors noticed that at temperatures above 400 °C, the unreacted residual Co underwent an allotropic phase transformation (from a hexagonal to cubic phase). Two allotropic phases of Mg_2_CoH_5_ (low- and high-temperature phases) were observed, with the change from the low-temperature to high-temperature phase occurring at approximately 200 °C. The authors suggested that no MgH_2_ phase was needed for Mg_2_CoH_5_ synthesis. According to them, the ternary hydride could be formed directly from pure elements under specific pressure and temperature conditions. Another breakthrough was made by Zepon et al. [277]. Nanocrystalline (particle size below 1 µm) Mg_2_CoH_5_ was synthesized from a 2Mg + Co mixture milled at 4 MPa hydrogen pressure for 3–24 h at room temperature. As a result, a 97% reaction yield was achieved. The absorption and desorption temperatures were estimated to be 300 and 350 °C, respectively. Increased temperature resulted in an increased hydrogen capacity and improved kinetics. The research also provided a broad description of the reaction mechanism. Similar to Mg_2_FeH_6_, the formation of the complex hydride Mg_2_CoH_5_ during RBM was a two-step reaction. First, Mg reacted with hydrogen, and a mixture of MgH_2_ and Co was formed. A further reaction path consisted of MgH_2_ reacting with Co and hydrogen and generating Mg_2_CoH_5_ as a result. The time needed to complete Mg_2_CoH_5_ formation was significantly shorter than that required for the Mg_2_FeH_6_ alloy with the same milling parameters [278]. Hydrogen desorption at low temperatures up to 325 °C was connected with a diffusion mechanism, and no change in the high-temperature crystalline phase of Mg_2_CoH_5_ occurred. Above that temperature, the high-temperature phase of Mg_2_CoH_5_ became unstable, and the ternary hydride decomposed into hydrogen and pure Mg and Co. Moreover, the DSC onset temperature for desorption of the reactive milled Mg_2_CoH_5_ was ~230 °C, which was approximately 50 °C lower than that in the Mg_2_FeH_6_ case. Moreover, nanocrystalline Mg_2_CoH_5_ synthesized by reactive milling presented considerably different hydrogen desorption behavior than Mg_2_FeH_6_.

**Figure 20:**
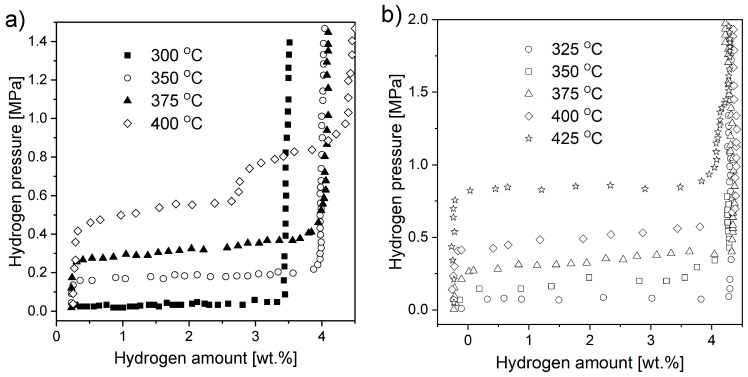
Desorption PCI curves of (**a**) a 2Mg-Co mixture milled in an argon atmosphere and (**b**) a 2Mg-Co mixture milled in a hydrogen atmosphere. The graphs are based on [279].

A comparison between Mg_2_CoH_5_ milled in an inert atmosphere and in a hydrogen atmosphere was performed by Veron et al. with 2Mg-Co and 2Mg-Co mixtures [279]. Three hydrides could be formed in the Mg-Co-H system under different conditions, i.e., Mg_6_Co_2_H_11_, Mg_2_CoH_5,_ and MgH_2_, which were observed as three plateaus in the pressure-composition isotherm (PCI) curves. The low-hydrogen-pressure plateau corresponded to Mg_6_Co_2_H_11_ formation, and the high-pressure plateau corresponded to Mg_2_CoH_5_. The decomposition enthalpy for the Mg_2_CoH_5_ phase was −84 kJ/mol H_2,_ and the hydride was almost unaffected by cycling. The desorption curves of samples milled in argon and hydrogen atmospheres are shown in Figure 20a,b, respectively. Unfortunately, no formation enthalpy data for the sample ball-milled in an argon atmosphere were provided from the information about hydrogen pressure after cycling. In contrast, the enthalpy value for the noncycled sample from BM was in good agreement with the value estimated for the sample synthesized in a hydrogen atmosphere.

### 5.2. Reaction Yield Analysis

**Figure 21:**
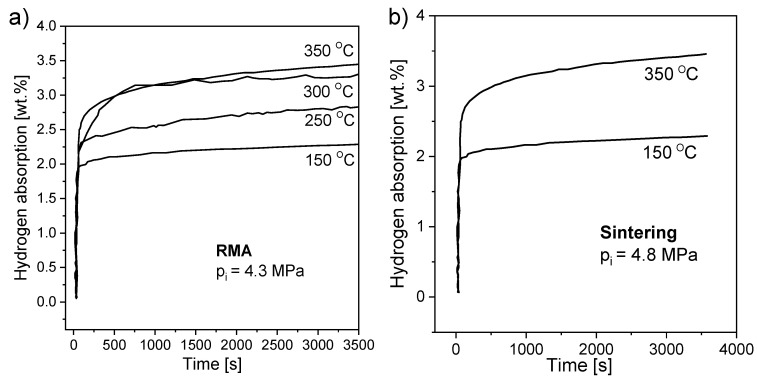
Absorption kinetics of Mg_2_CoH_5_ produced by (**a**) RMA and (**b**) sintering at different pressures. The graphs are based on [33].

A microstructural analysis of the Mg_2_CoH_5_ alloy produced by RBM (0.4 MPa H_2_) of a 2Mg-Co mixture was performed by Gennari et al. [34]. High reactivity (compared with that of Mg_2_FeH_6_ ball milled under the same conditions [38]), i.e., a high yield with short milling times, was achieved due to improved intermixing and contact between the powders. An endothermic peak indicating hydride decomposition was observed at ~300 °C independent of milling time. From XRD and DSC analysis, the amount of Mg_2_CoH_5_ was estimated as 20 wt.% after 90 h of milling. Fernandez et al. [33] combined mechanical milling and MA. A 2Mg-Co mixture was milled (200 h) in an argon atmosphere and then milled (90 h) in a hydrogen atmosphere, both at room temperature. As a result, a 50% synthesis yield was achieved. Premilling caused microstructural refinement and Mg-Co intermixing, which influenced the kinetics of Mg_2_CoH_5_ formation. The elemental Co phase acted as a catalyst for hydrogen dissociation and association. Hence, the sorption kinetics rate increased. Cycling tests revealed that the desorption onset temperature changed due to the nonuniform grain size. Two plateaus were observed, which indicated the presence of two phases, Mg_2_CoH_5_ and Mg_6_Co_2_H_11_. The decomposition peak was observed at 205 °C. Kinetics measurements were performed at a constant temperature between 150 and 350 °C (Figure 21a). The best hydrogen capacity was measured at 350 °C (~3.5 wt.%), which was in good agreement with the results for sintered samples (Figure 21b). A great advantage of the RMA technique over the sintering technique is the fact that the produced complex hydride is characterized by very fast absorption (70% of the overall hydrogen uptake occurred before 100 s) because of more uniform grains and their refinement after cycling. On the other hand, a nonuniform grain size was observed for the sintered samples, which influenced the onset desorption temperature.

**Figure 22:**
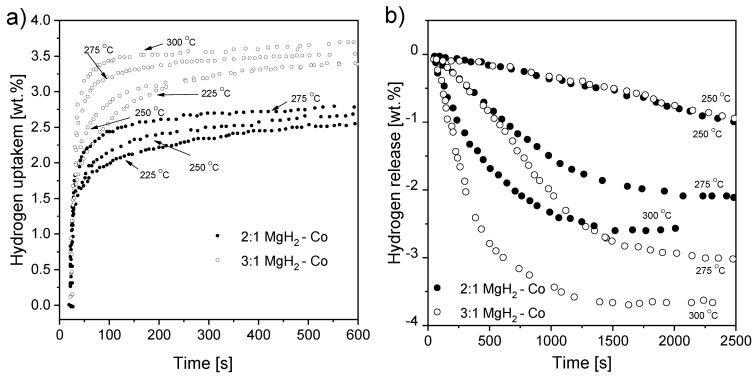
(**a**) Hydrogen absorption curves at various temperatures after 5 h of RMM at a 2 MPa initial pressure and (**b**) hydrogen desorption curves after 5 h of RMM at a hydrogen desorption pressure of 0.02 MPa. The graphs are based on [280].

Mg_2_CoH_5_ was synthesized by single-step RMM in a hydrogen atmosphere (0.5 MPa) from 2MgH_2_-Co and 3MgH_2_-Co mixtures [280]. With the 2Mg-Co mixture, a yield of 83% was achieved with an assumed ΔH = 82 kJ/mol H_2_. The measured volumetric hydrogen desorption was 3.8 wt.%, which was relatively close to the theoretical value (4.4 wt.%). No endothermic peak associated with transformation from a tetragonal to cubic structure (~210 °C) was observed, which may be caused by the difficulty of detecting the phase transition due to the nanometric grain size, as noted before [33,34]. Even with the 3MgH_2_-Co mixture, Mg_2_CoH_5_ was the dominant phase, with a small amount of Mg_6_Co_2_H_11_ as a secondary phase (at high temperature and pressure). Hence, the reaction yield was ~80%. Additionally, Mg_2_CoH_5_ had the highest hydrogen storage capacity (3.7 wt.%) between 250 and 300 °C. Hydrogen absorption and desorption curves for different MgH_2_-Co ratios and different temperatures are visible in Figure 22a,b. The formation of Mg_2_CoH_5_ from a 3MgH_2_-Co mixture proceeds by not fully and symmetrical reversible reaction, which is why Mg_2_CoH_5_ is not appropriate for mobile applications.

BM and ball alloying are possible alternatives for synthesizing hydrides with good outcomes. However, as always, the above techniques and processes need to be better understood and optimized. Outstanding performances were reported by Norek et al. [276] and Zepon et al. [277] with reaction yields of ~90% and 97%, respectively.

### 5.3. Cycling Stability

Much research has shown different estimated enthalpy values of magnesium-cobalt hydride. As mentioned before, the first attempt was performed by Ivanov et al. [272], who obtained −79 and −70 kJ/mol H_2_ for the hydrogen desorption of the Mg_2_CoH_5_ and Mg_3_CoH_5_ phases, respectively. Later, the Mg_2_CoH_5_ enthalpy values were estimated as −95 kJ/mol H_2_ and −108 kJ/mol H_2_ for the lower and upper plateau, respectively [281]. Zolliker et al. [271] published different values for the heat of dissociation (86 kJ/mol H_2_) and heat of formation (60 kJ/mol H_2_) of the Mg_2_CoH_5_ phase. The abovementioned works prove that the hydrogen loading and cycling stability of the Mg_2_Co-H hydride system have been examined broadly. Unfortunately, the results are not in good agreement, and some issues remain unsolved (especially the effect of BM on ternary hydride properties).

**Figure 23:**
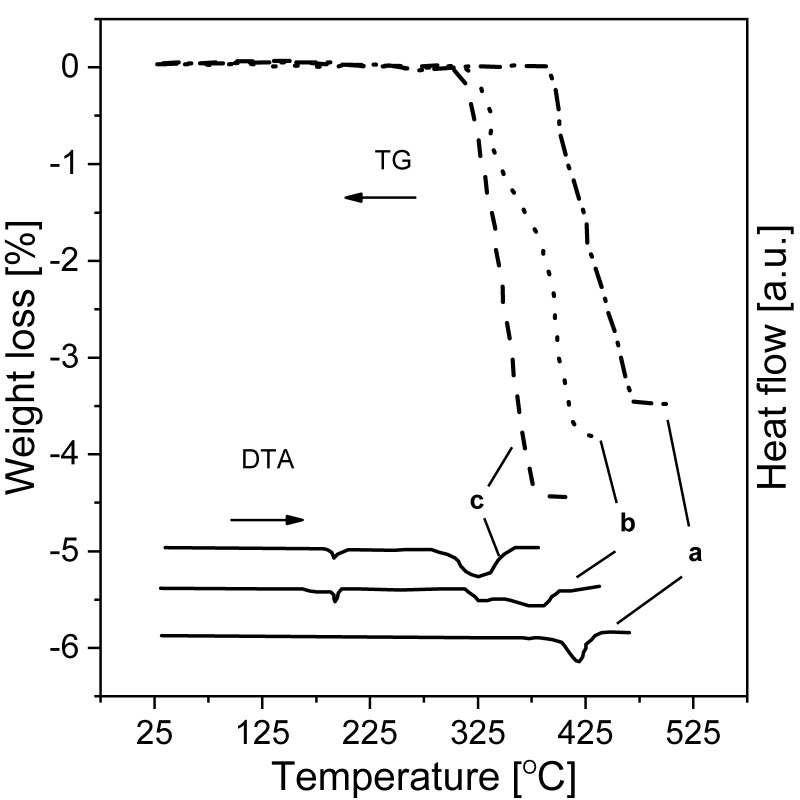
DTA and TG curves of (a) the initial 2MgH_2_ + Co mixture and ball-milled sample and after (b) 2 h and (c) 10 h. The graph is based on [281].

The synthesis of Mg_2_CoH_5_, a nanocrystalline complex metal hydride, was performed by MA for the first time by Chen et al. in 2001 [281]. A mixture of 2MgH_2_ + Co was mechanically alloyed in a hydrogen atmosphere. The hydrogenation characteristics were investigated and indicated a noted improvement in the kinetics of hydride formation. As a result of the very fine microstructure obtained by BM (the powder had a dark gray color), the decomposition temperatures for MgH_2_ and Mg_2_CoH_5_ shifted to lower values. The Mg_2_CoH_5_ dissociation and formation enthalpy values were −83.2 and 69.5 kJ/mol H_2_, respectively. More rapid hydride formation was possible due to the presence of milled cobalt, which influenced fast hydride formation via a metastable phase. It could be concluded from Figure 23 that with increasing BM time, the hydrogen content of the sample also increased, and the dehydrating reactions shifted to a lower temperature. This result was attributed to the kinetic behavior of the powder, which was a consequence of changes in the surface characteristics of the milled powders. After 20 cycles, a 2% hydrogen capacity degradation was observed. These results do not compare well with [279,280], where the hydrogen equilibrium pressure and hydrogen storage capacity were stable upon cycling.

Reiser et al. [204] carried out a broad investigation of the cyclic stability of a Mg-Co-H system for two ternary hydrides: Mg_2_CoH_5_ and Mg_6_Co_2_H_11_. The material was still considered as a good heat/energy storage system despite differences in its thermodynamic data. Excellent stability of the obtained hydride was observed after more than 1000 cycles. Two plateaus existed in the region between 2.5 and 3.7 wt.% hydrogen content. The equilibrium pressure for the first region was calculated and corresponded to the heat of desorption, equal to 76 kJ/mol H_2_. For the second region, no reliable value could be calculated. Nevertheless, the relatively high enthalpy of formation made this system sufficient as a heat storage medium. The dissociation pressure for the first plateau was lower than those for the previously discussed Mg-Fe-H and Mg-Ni-H systems.

## 6. General Conclusions and Future Prospects

Despite the likely thousands of extremely interesting papers published in the literature and presented to some extent in this review, magnesium hydride and magnesium-based hydrides will likely not solve the hydrogen storage problem, especially for mobile applications. A relatively high gravimetric capacity of the material itself is likely not enough to overcome the low practical (despite very high theoretical) volumetric capacity and problems related to handling and reactivity. Consequently, other, more “friendly” and easy-to-use materials are likely to be applied in practice. However, the fact that magnesium hydrides and magnesium-based hydrides have been investigated for decades now and are still not fully understood proves that there is still significant potential in this type of material in terms of the basic science and from a theoretical standpoint, as well as for other applications (such as electrodes for batteries). Thus, there is still much to be discovered and confirmed. Despite the fact that we are skeptical about the potential mobile applications and hydrogen storage capability of magnesium hydrides and magnesium-based hydrides, there is significant practical potential in these materials for waste heat storage in the temperature range of 400–550 °C due to their high enthalpy values of formation and decomposition.

**Figure 24:**
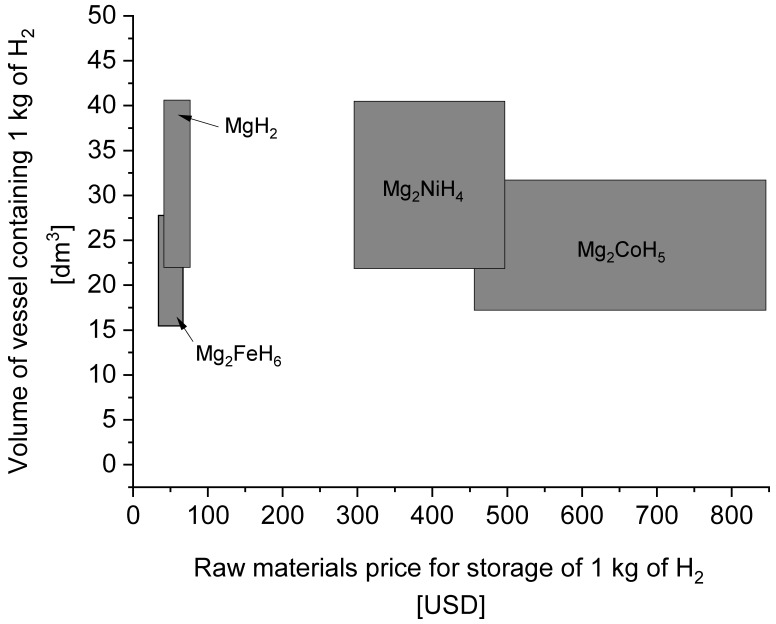
Graph showing the volume of the tank and cost of the powder required to store 1 kg of hydrogen in the form of the chosen compound.

Among all the problems shown in this review, such as synthesis yield issues, kinetics issues, cyclic stability issues, thermodynamic stability, degradation etc., one much less scientific problem is of the greatest importance—surprisingly, the cost issue. Not mentioned too much in the literature, among all those described materials having quite similar properties, only two seem to have a real application potential due to their acceptable price. Figure 24 shows the comparison of the four compounds described in this review in terms of their hydrogen storage effectiveness by volume and price. Each of them is represented by the area on the graph since both the possible yield and the bulk density of the powders were considered. It is more than clear that while having very similar volumetric densities, and also quite similar working temperatures (not shown here), both Mg_2_NiH_4_ and Mg_2_CoH_5_ are definitely too expensive to consider them for practical use while MgH_2_ and Mg_2_FeH_6_ are available.

There are, however, methods to make the production of such storage systems cheaper. In particular, using scrap materials as substrates to lower the cost of production may be beneficial. However, even if the price will be acceptable if these materials are to be applied for high-volume, high-risk storage, all the unknowns must be solved in advance to avoid technical difficulties or even potential material-related failures and tragedies.

A big problem in describing those materials in the form of a review paper has probably also been spotted by readers of this review, and it lies far from any physical or chemical property. The great number of experiments performed within this topic have not, in our opinion, resulted in obtaining general, systematic knowledge about the behavior of these materials. There are several factors causing this deficiency, but the most important, in our opinion, are the lack of standard conditions and procedures for testing this group of materials, imprecise experimental descriptions, and a lack of standard examination of possible impurities in the investigated systems. For these reasons, the results in most cases can be compared only qualitatively. One of the biggest challenges for the hydrogen storage society is to normalize the measurement conditions to such an extent that newly discovered materials can be easily compared with the old ones.

## Figures and Tables

**Table 1:** Catalytic effect of different additives on the properties of some BM-synthesized Mg-based hydrides.

Material	Temperature (°C)	Pressure (MPa)	Max. H_2_ (wt.%)	Approx. Absorption Time (s)	Approx. Desorption Time (s)	Reference	Remarks
MgH_2_	T_abs_ = 200 T_des_ = 300	p_abs_ = 1 p_des_ = 0.015	4.7	1000	1000	[85]	Kinetics curves of the samples measured on the third cycle
MgH_2_-5 at.% Ti	T_abs_ = 200 T_des_ = 300	p_abs_ = 1 p_des_ = 0.015	5.0	50	200		
MgH_2_-5 at.% V	T_abs_ = 200 T_des_ = 300	p_abs_ = 1 p_des_ = 0.015	5.5	50	300		
MgH_2_-5 at.% Mn	T_abs_ = 200 T_des_ = 300	p_abs_ = 1 p_des_ = 0.015	6.0	200	500		
MgH_2_-5 at.% Fe	T_abs_ = 200 T_des_ = 300	p_abs_ = 1 p_des_ = 0.015	4.3	200	300		
MgH_2_-5 at.% Ni	T_abs_ = 200 T_des_ = 300	p_abs_ = 1 p_des_ = 0.015	4.7	1000	400		
MgH_2_ + (Cr_2_O_3_)_0.05_	T_abs_ = 300	p_abs_ = 1.5	4.02	600	Only absorption kinetics was measured	[86]	High-energy BM
MgH_2_ + (V_2_O_5_)_0.05_	T_abs_ = 250	p_abs_ = 1.5	3.2	600			
MgH_2_ + (Al_2_O_3_)_0.05_	T_abs_ = 300	p_abs_ = 1.5	4.09 4.49	600 4000			
MgH_2_ + (Fe_2_O_3_)_0.05_	T_abs_ = 300	p_abs_ = 1.5	1.37 3.53	600 5000			
Nano − MgH_2_	T_abs_ = 300	p_abs_ = 0.8	5.97	178	Only absorption kinetics was modeled	[87]	Modeling of the hydriding kinetic properties of a nanocomposite
MgH_2_ + (Cr_2_O_3_)_0.05_	T_abs_ = 300	p_abs_ = 0.8	4.64	32			
MgH_2_ + (V_2_O_5_)_0.05_	T_abs_ = 300	p_abs_ = 0.8	4.07	39			
MgH_2_ + (Fe_2_O_3_)_0.05_	T_abs_ = 300	p_abs_ = 0.8	4.31	35			
MgH_2_ + 0.5 mol.% Nb_2_O_5_	T_abs_ = 250 T_des_ = 300	p_abs_ = 0.84 p_des_ = 0.84	7.0	60	90	[88]	MgH_2_ ball milled for 20 h and later combined with Nb_2_O_5_
MgH_2_ + 1 mol.% Nb_2_O_5_	T_abs_ = 150 T_des_ = 160	p_abs_ = 0.1 p_des_ = 0.1	6	30	60,000	[89]	Desorption in a purified helium flow atmosphere with zero hydrogen partial pressure
MgH_2_ + 1 mol.% La_2_O_3_	T_abs_ = 300 T_des_ = 300	p_abs_ = 0.3 p_des_ = 0.03	6.0	100	430	[90]	Samples annealed at different temperatures
